# Regulating Li^+^ Transport and Interfacial Stability with Zwitterionic COF Protective Layer Towards High-Performance Lithium Metal Batteries

**DOI:** 10.1007/s40820-025-02017-3

**Published:** 2026-01-05

**Authors:** Liya Rong, Yifeng Han, Chi Zhang, Hongling Yao, Zhaojun He, Xianbao Wang, Zaiping Guo, Tao Mei

**Affiliations:** 1 Hubei Collaborative Innovation Center for Advanced Organic Chemical Materials, Overseas Expertise Introduction Center for Discipline Innovation (D18025), Key Laboratory for the Green Preparation and Application of Functional Materials, Hubei Key Laboratory of Polymer Materials, College of New Energy and Electrical Engineering, Hubei University, Wuhan, 430062 People’s Republic of China; 2 Department of Materials Science and Engineering, City University of Hong Kong, Kowloon, Hong Kong, 999077 People’s Republic of China

**Keywords:** Zwitterionic covalent organic framework, Li^+^ migration kinetic regulation, Li^+^ desolvation, Charge distribution, Interface stability

## Abstract

The sluggish Li^+^ migration kinetics and unstable electrode/electrolyte interface severely hinder the commercial application of high-performance lithium metal batteries (LMBs). Herein, an artificial protective layer is constructed using zwitterionic covalent organic framework (Z-COF) simultaneously containing sulfonate and ethidium groups, aiming to facilitate rapid, uniform Li^+^ transport and stabilize anode interface. The sulfonate groups with high lithiophilicity provide abundant hopping sites for fast Li^+^ diffusion. The ethidium cations immobilize TFSI^−^ and solvent molecules by ion–dipole interactions, which accelerate the dissociation of LiTFSI and Li^+^ desolvation. Moreover, the monodispersed zwitterionic units coupling with ordered micropore structures in Z-COF create exclusive Li^+^ migration channels, modulate homogeneous space charge distribution, kinetically facilitating uniform Li^+^ deposition. Experiments and theoretical calculations indicate that C–F and S–N bonds of TFSI^−^ exhibit enhanced cleavage susceptibility driven by electrostatic attraction, realizing a LiF/Li_3_N-rich electrolyte/electrode interface. The designed Z-COF protection layer enables Li|Li symmetrical cells stable cycling over 6300 h at 2 mA cm^−2^/2 mAh cm^−2^. The Z-COF@Li|LiFePO_4_ (LFP) full cells deliver high-capacity retention of 85.2% after 1000 cycles at 8 C. The assembled Z-COF@Li|LFP pouch cells demonstrate a lifespan of more than 240 cycles. This work provides fresh insights into the practical application of zwitterionic COF in next-generation LMBs.

**Supplementary Information:**

The online version contains supplementary material available at 10.1007/s40820-025-02017-3.

## Highlights

- Ethidium cations acted as “anion capturers” to immobilize TFSI^−^, which rendered the C-F and S-N bonds prone to cleavage, facilitating the formation of LiF/Li3N-rich solid electrolyte interphase.
- Ion–dipole interaction between ethidium groups and dimethoxyethane/dioxolane, boosting Li^+^ desolvation.
- Sulfonate groups exhibited an ion-sieving effect that selectively attracted Li⁺ while excluding TFSI⁻, promoting LiTFSI dissociation and accelerating Li^+^ migration.

**Supplementary Information:**

The online version contains supplementary material available at 10.1007/s40820-025-02017-3.

## Graphical abstract

**Figure:**
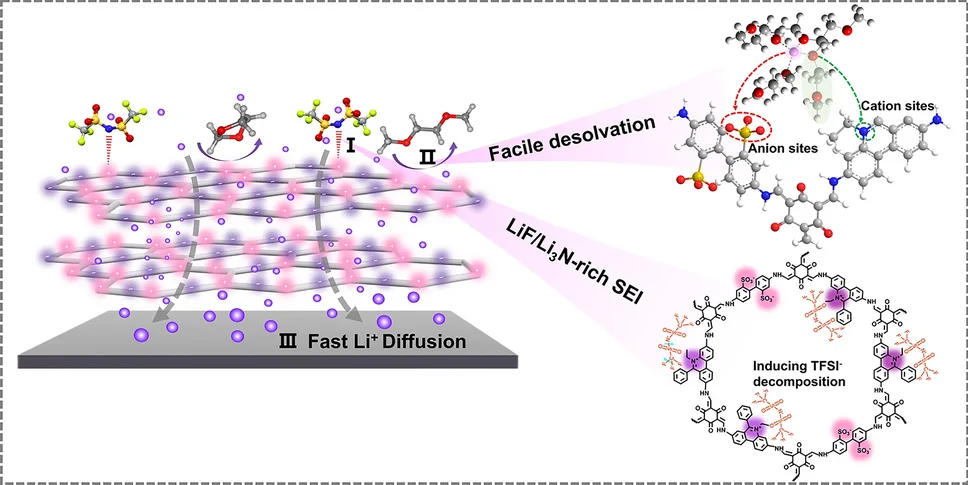

## Introduction

Commercial energy storage devices cannot satisfy ever-rising demands for high-end communication terminals and electric vehicles because of their theoretical energy density limits [1–5]. The development of high-energy-density rechargeable batteries has become an urgent problem. Lithium metal batteries (LMBs) are considered promising competitors in this pursuit, owing to ultrahigh theoretical specific capacity of 3860 mAh g^−1^ and the lowest electrochemical potential (− 3.04 V vs. standard hydrogen electrode) [6–9]. However, uncontrollable Li dendrite growth induces poor Coulombic efficiency (CE), irreversible capacity loss and severe safety hazard, dragging LMBs out of practical applications [10, 11]. According to the Sand’s equation, interfacial Li^+^ migration significantly influences Li electrodeposition process [12–14]. The sluggish Li^+^ diffusion can lead to the concentration polarization between bulk electrolyte and anode surface, fostering uneven Li plating/stripping [15–18]. In addition, the nonuniform accumulation of free anions leads to an uneven charge distribution near the electrode surface, exacerbating the growth of Li dendrites [19, 20]. Hence, it is imperative to develop a collaborative strategy which simultaneously modulates the ion interface kinetics transfer and local charge distribution to induce rapid and uniform Li deposition for realizing high-performance LMBs.

As an emerging crystalline porous material, ionic covalent organic framework (iCOF) can greatly present both crystal and ionization characteristics, garnering widespread attention in Li^+^ transportation [21–25]. The ordered open nanochannels of iCOF can serve as fast Li^+^ conduction pathway [26–29]. The permanent charged ion units on pore walls capable of interacting with Li^+^ or anions grant iCOF outstanding Li^+^ selectivity [30–33]. Recently, various reports declared that single anionic COF could effectively inhibit free anions (TFSI^−^ and PF_6_^−^) transport while accelerating Li^+^ migration owing to surface-negative charge-induced ion screening effect [34, 35]. Besides, the single cationic COF could alleviate the degree of Li^+^ solvation by the intermolecular interaction between the positive charge groups and the solvent molecules, thereby regulating Li^+^ desolvation process [36–38]. However, single iCOF-modified strategy focused solely on Li^+^ diffusion or desolvation behaviors, addressing only a portion of the Li^+^ transfer issues. Engineering COF with zwitterionic units and fully exploiting the functionality of cationic/anionic ion sites may offer additional perspectives for overcoming the above challenges.

Zwitterionic COF possesses mono-dispersed ion moieties with both cationic and anionic units, but still maintains overall charge neutrality [39–41]. This structure feature endows zwitterionic COF with higher dipole moments, easier tunable charge density, more abundant ion adsorption/migration sites and superior ion-conductive property beyond single iCOF [42, 43]. Recently, a locally zwitterionic covalent organic framework nanosheets (ziCOFNs) containing triaminoguanidinium cation and carboxylate anion were developed as Li^+^ accelerated regulators to promote Li^+^ migration and stabilize interface chemistry of lithium metal anodes (LMAs) [44]. Another zwitterionic COF (Zwitt-COF) involving pyridinium cation and carboxylate anion was prepared as a solid electrolyte to achieve an improved dissociation and transport of Li^+^ [45]. These distinguished research endeavors demonstrate that the customization of anionic and cationic groups in zwitterionic COF can significantly improve Li^+^ transport properties. To maximize the synergistic effects of both cationic and anionic moieties in regulating Li^+^ migration kinetics, the design strategy of zwitterionic COF should adhere to the following key principles: The cationic moieties can effectively capture anions/solvent molecules to accelerate dissociation of ion pairs and desolvation of Li^+^. The anionic groups should possess Li⁺ affinity, functioning as lithiophilic sites to concentrate Li^+^ along the COF nanochannels, thereby accelerating Li^+^ conduction.

**Fig. 1:**
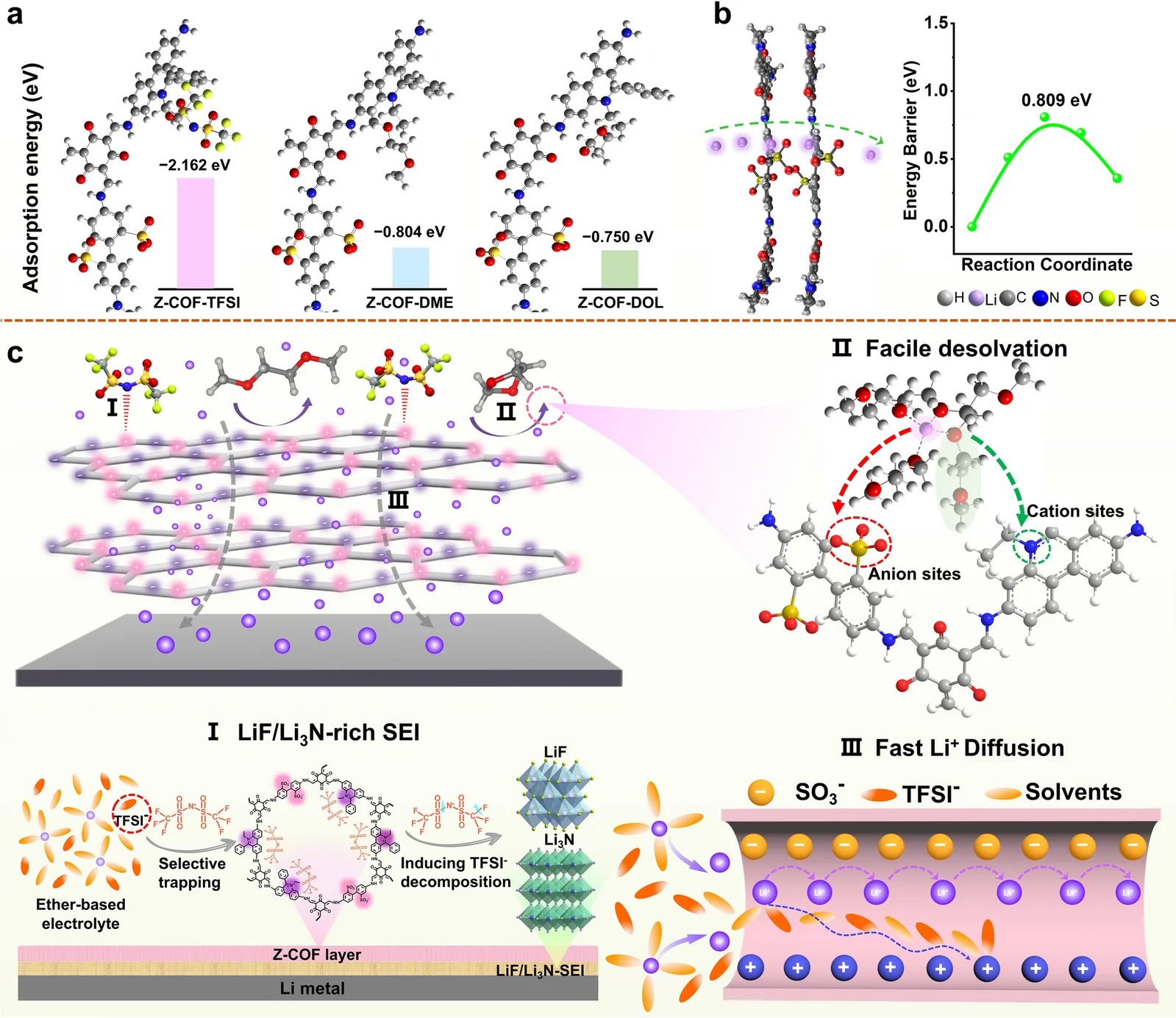
**a** Chemical coordination circumstance of simulated adsorption energy between Z-COF fragment and LiTFSI-based electrolyte with the terminal optimized geometries. **b** Theoretical calculation of Li^+^ migration along the axial orientation inside Z-COF and the corresponding migration energy barrier. **c** Mechanism description of Z-COF protective layer to induce the formation of LiF/Li_3_N-rich SEI, facilitate Li^+^ desolvation and boost Li^+^ migration

Guided by these principles, a zwitterionic COF simultaneously containing sulfonate and ethidium groups (denoted as Z-COF) was rationally designed as a protective layer on LMAs surface to regulate Li^+^ migration kinetics, solid electrolyte interphase (SEI) components evolution and interface charge distribution. The calculated negative adsorption energy values (Fig. 1a) based on density functional theory (DFT) indicated that ethidium groups could trap anions (TFSI^−^, − 2.162 eV) and solvent molecules (dimethoxyethane/dioxolane, DME/DOL, − 0.804/ − 0.750 eV) in the electrolyte. Specifically, the ethidium groups could act as “anion capturers” to immobilize TFSI^−^, thus promoting LiTFSI dissociation. The ion–dipole interaction ensured the anchoring of ethidium cations to DME/DOL and was conducive for the liberation of Li^+^ from the solvent clusters, boosting Li^+^ desolvation. The ion-selective transport behavior enabled sulfonate groups to attract Li⁺ while repelling TFSI^−^, providing additional driving force for LiTFSI dissociation and dominating kinetically enhanced Li^+^ migration toward pore walls of Z-COF. As shown in Figs. 1b, S1 and S2, the lower Li^+^ migration energy barrier (0.809 eV), compared to that of the corresponding single iCOF (BDSA-COF: 1.271 eV, EB-COF:1.718 eV), demonstrated that Li^+^ could preserve fast diffusion within Z-COF channels. It was attributed to the sulfonate groups exhibited a larger Li⁺ dissociation constant, giving rise to a higher Li⁺ conductivity compared with other anionic groups such as phenolate and carboxylate [46]. In addition, the innate steric bulkiness of sulfonate groups tended to form continuous ionic clusters between adjacent COF layers, providing abundant hopping sites for rapid Li^+^ diffusion. More importantly, the capture effect of ethidium cations induced elongation of the C–F and S–N bonds in TFSI^−^ and rendered the corresponding chemical bonds prone to cleavage, thereby facilitating the formation of a LiF/Li_3_N-rich SEI layer (Fig. 1c). Finally, the monodispersed ethidium and sulfonate charged units integrated with ordered channels of Z-COF guaranteed the formation of uniform local electric fields on the LMAs surface and homogenized Li^+^ flux, efficaciously restricting the Li dendrites growth. As a result, Z-COF-modified symmetrical cells realized an ultra-long cycling life of more than 6300 h at 2 mA cm^−2^/2 mAh cm^−2^. The Z-COF@Li|LiFePO_4_ (LFP) full cells displayed a pro-longed cycling life over 1000 cycles with capacity of 92.1 mAh g^−1^ at 8 C even under the harsh conditions (LFP mass loading: 8.76 mg cm^−2^, electrolyte: 2.3 μL mg^−1^). The assembled Z-COF@Li|LFP pouch cells demonstrated a lifespan of more than 240 cycles without obvious voltage fluctuation.

## Experimental Section

### Synthesis of Z-COF

In a typical solvothermal methods, ethidium bromide (EB, 0.2 mmol) and 4,4'-diaminobiphenyl-2,2'-disulfonic acid (BDSA, 0.1 mmol), along with 3 mL of the mixture of 1,4-dioxane and mesitylene (v/v, 1:1), were added to a Pyrex tube. After sonicating this tube for 20 min to fully mix the two amine monomers, this tube was charged with 1,3,5-triformylphloroglucinol (Tp, 0.2 mmol) and 0.6 mL of 12 M aqueous acetic acid. After sonication for 10 min, the tube with reaction mixture was then flash frozen at 77K and degassed by three freeze–pump–thaw cycles. The tube was sealed and then heated at 120 °C for 5 days. After cooling process, a dark red precipitate was collected by filtration and washed with DMF and water for several times, followed by a Soxhlet extracting with ethanol and methanol to solvent exchange, and dried at 120 °C under vacuum overnight to get corresponding Z-COF in ~ 82% isolated yield.

### Fabrication of Electrode

#### Fabrication of COF@Li Electrode

1 mg Z-COF powder was dispersed in 1 mL THF and stirred for 12 h to form 1 mg mL^−1^ uniform suspension solution. Then, the 30 µL suspension solution was dripped on the Li surface (*φ* = 15.6 mm). Subsequently, it was dried under the glove box at room temperature for 12 h until the solvent is completely evaporated.

#### Fabrication of COF@Cu Electrode

The Z-COF@NMP suspension solution was fabricated through mixing Z**-**COF composite, binder (PVDF) together with a reasonable mass ratio of 4:1, followed by coating onto each Cu foil (*φ* = 12 mm) for 30 μL. Then the Z**-**COF@Cu was obtained after drying in vacuum (60 °C, 12 h).

#### Fabrication of LFP Cathode

The LFP cathode was prepared by a typical tape-casting method. Commercialized LFP powder, super P and PVDF binder (7:2:1 in weight) were mixed in NMP solution to form a homogeneous slurry. Then the slurry was scraped onto the carbon-coated aluminum foil by a blade and dried at 100 °C overnight under vacuum. Finally, the cathode-coated Al foil was cut into disks with a diameter of 12 mm. The mass loading could be controlled by adjusting the coating thickness.

## Results and Discussion

### Fabrication and Characterizations of Z-COF

**Fig. 2:**
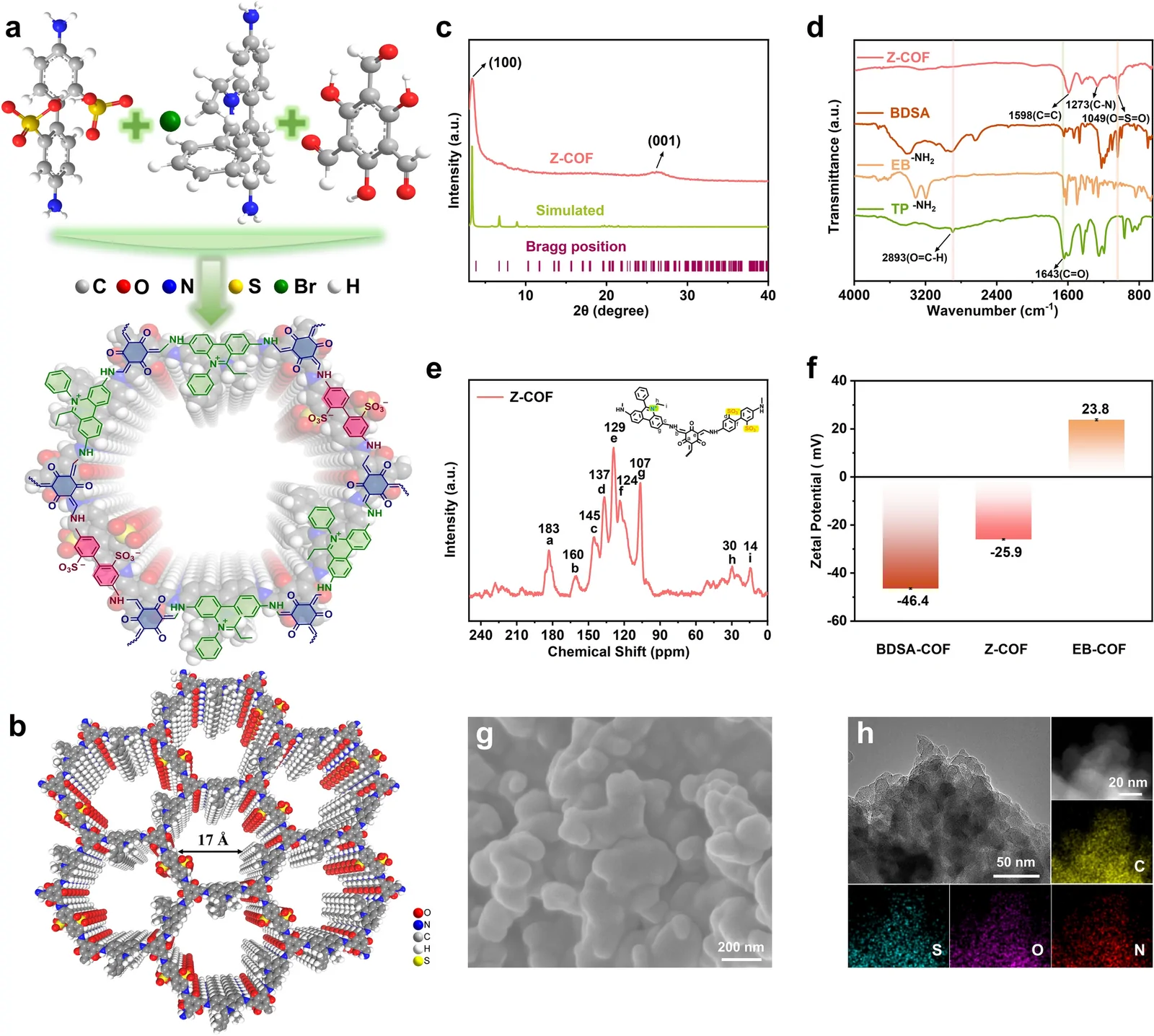
Structure and morphology characterizations of Z-COF. **a** Reaction scheme for the synthesis of Z-COF. **b** Top and side views of graphic presentations of Z-COF. **c** Experimental and simulated PXRD patterns of Z-COF. **d** FT-IR spectra of Z-COF. **e** ^13^C solid-state NMR spectrum of Z-COF. **f** Zeta potential of Z-COF and the corresponding anionic/cationic COFs. **g** SEM image of Z-COF. **h** HR-TEM and related EDX elemental mapping images of Z-COF

The zwitterionic COF (Z-COF) was obtained by molar ratio (2:1:2) of 4,4'-diamino-2,2'-stilbenedisulfonic acid (BDSA), ethidium bromide (EB) and 1,3,5-triformylphloroglucinol (Tp) in a mixed solvent system consisting of 1,4-dioxane, mesitylene and 12 M aqueous acetic acid (v/v/v, 5:5:2) at 120 °C for 5 days according to Schiff base condensation reaction (Fig. 2a) [47]. Interlayer π-π stacking interactions in the Z-COF promote the formation of aligned nanochannels (17 Å) and extendable topological structure (Fig. 2b). The crystallinity of Z-COF was verified by powder X-ray diffraction (PXRD) and simulated patterns. As shown in Fig. 2c, the diffraction peaks for Z-COF at 3.4° and 26° were assigned to the (100) and (001) facet, respectively, corroborated by structural simulations, verifying intact crystallinity of the obtained samples. The Fourier transform infrared (FT-IR) spectroscopy was used for investigating chemical structure of Z-COF (Fig. 2d), the absence of characteristic peaks of the N–H stretches (3100–3400 cm^−1^) of NH_2_ groups in the EB/BDSA and the aldehyde group stretches (O=C–H: 2893 cm^−1^, C=O: 1643 cm^−1^, respectively) of TP corroborated the occurrence of Schiff base polycondensation. Besides, the disappearance of imine (C=N) stretching peak (1620 cm^−1^) and the appearance of new C=C (1598 cm^−1^) and C–N (1273 cm^−1^) peaks confirmed that the keto form existed in Z-COF. The stretching peak at 1049 cm^−1^ was attributed to sulfonate groups in Z-COF. The peak at 183 ppm further revealed the presence of the keto form in the cross-polarization magic angle spinning ^13^C solid-state NMR (^13^C SS NMR) spectrum (Fig. 2e). Additional signals at 14–30 ppm corresponded to ethyl carbons, indicating the existence of EB monomer in the skeleton. Furthermore, the peak at 137 ppm was ascribed to the carbon atom in the C–S bond, certifying the existence of sulfonate.

The high-resolution X-ray photoelectron spectroscopy (XPS) analysis verified that the element compositions of Z-COF were C, N, O, and S (Fig. S3a**)**. The peaks at 399.6 and 401.3 eV were assignable to free secondary amine from –NH– linker and the pyridinium N from EB monomer in the deconvoluted N 1*s* XPS spectra (Fig. S3b), respectively. Quantitative analysis showed a 3:1 ratio of two different types of nitrogen. The measured N/S ratio (3.6:1) closely matched the theoretical 4:1 value, demonstrating near-ideal zwitterionic pairing in the Z-COF skeleton (Table S1). The result of elemental analysis matched well with the theoretical calculation values (Table S2) further validated the stoichiometric incorporation of both cationic and anionic components. The porous nature of Z-COF was quantitatively assessed by N_2_ adsorption isotherm measurement at 77 K (Fig. S4a). Typical type-I isotherm was observed, indicating Z-COF exhibited microporous structure. The Brunauer–Emmett–Teller (BET) surface area was calculated to be 303.87 m^2^ g^−1^. As estimated by nonlocal density functional theory (NLDFT), the pore size distribution of Z-COF was 16.8 Å, which was consistent with theoretical pore size. The thermogravimetric analysis (TGA) revealed that Z-COF possessed excellent thermal stability, up to 270 °C in N_2_ atmosphere (Fig. S4b). Additionally, we observed that the supernatant liquid of Z-COF remained clear and crystalline structure maintained unchanged after storage in a LiTFSI-based electrolyte for three weeks, suggesting the insolubility and superior stability of Z-COF in the electrolyte (Fig. S5a, b). The Z-COF protective layer on the LMAs could be preserved well with no structural collapse after 50 cycles (Fig. S5c), confirming that the superior structural stability of Z-COF during cycling.

Surface charge property of Z-COF was evaluated by zeta potential measurements. As illustrated in Fig. 2f, the average zeta potential of Z-COF was about − 25.9 mV, which located in between the cationic EB-COF (+ 23.8 mV) and anionic BDSA-COF (− 46.4 mV). These results showed that the surface of Z-COF was loaded with zwitterionic charged units. The dye adsorption experiment was conducted to visually validate the charged state on the surface of Z-COF. As demonstrated in Fig. S6, Z-COF and corresponding cationic/anionic COFs (EB-COF and BDSA-COF) were soaked in methylene blue (positively charge), nile red (charge–neutral) and methyl orange (negatively charged) solutions for 24 h, respectively. The cationic EB-COF only captured methyl orange and the anionic BDSA-COF selectively adsorbed methylene blue, demonstrating complementary charge affinity. Unlike single-charged COFs, Z-COF exhibited effective adsorption toward both cationic and anionic dyes, verifying its zwitterionic character with balanced positive/negative sites.

Scanning electron microscopy (SEM) analysis (Fig. 2g) displayed that Z-COF presented as nanosphere. The energy-dispersive X-ray spectroscopy (EDX) elemental mapping (Fig. 2h) showed the uniform distribution of C, N, O, and S elements in Z-COF. High-resolution transmission electron microscopy (HR-TEM) images exhibited apparent lattice fringes (Fig. S7); the lattice spacing of ~ 3.6 Å was obtained by the inverse fast Fourier transformation, sufficiently proving a high crystallinity of Z-COF.

### Lithium Deposition and Stripping Behaviors

**Fig. 3:**
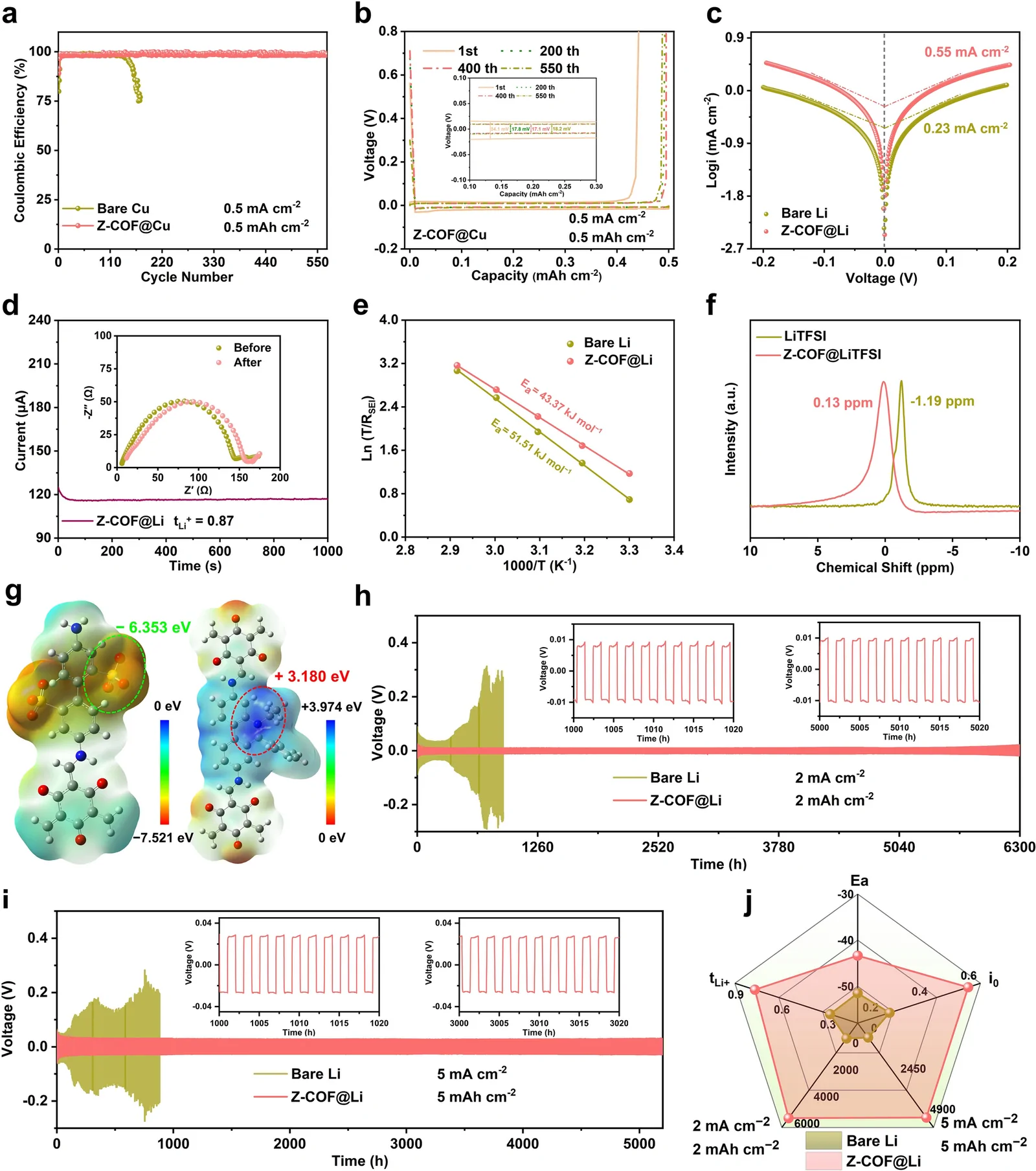
**a** Coulombic efficiency of the Li|Cu half cells at 0.5 mA cm^−2^/ 0.5 mAh cm^−2^. **b** Voltage–capacity curves of Li|Z-COF@Cu half cells (inset: partial enlarged detail). **c** Tafel plots of Li|Li symmetrical cells. **d** Li^+^ transference number (t_Li_^+^) of Z-COF@Li symmetrical cells (inset: Nyquist plots of impedance before and after polarization). **e** Arrhenius plots and activation energy of the symmetrical cells with or without Z-COF protective layer. **f** ^7^Li solid-state NMR spectra of LiTFSI and Z-COF-soaked LiTFSI-based electrolyte. **g** Electrostatic potential mapping of Z-COF. **h** and **i** Galvanostatic cycling of Li|Li symmetrical cells at 2 mA cm^−2^/2 mAh cm^−2^ and 5 mA cm^−2^/5 mAh cm^−2^, respectively. **j** Radar chart of electrochemical performance of Li|Li symmetrical cells

To evaluate lithium metal utilization, Coulombic efficiency measurements were taken on Li|Cu asymmetric cells during continuous cycling. As shown in Fig. 3a, the half cells assembled with Z-COF@Cu exhibited a stable CE over 99.65% after 550 cycles at 0.5 mA cm^−2^/ 0.5 mAh cm^−2^. Terribly, the bare Cu only afforded 140 cycles with a dramatic drop of CE under the identical current density. The voltage–capacity curves of Li|Cu cells are also plotted in Figs. 3b and S8a. Apart from the initial voltage hysteresis (VH) of Z-COF was 34.1 mV, and then the VH maintained at about 17–18 mV as the cycle went on, demonstrating the Z-COF protective layer performed a superior interfacial stability during the plating/stripping process. Bare Cu electrode displayed enormous voltage fluctuations, which were 55.4, 24.2, 35.3, and 28.8 mV at 1st, 50th, 100th, and 140th cycles, respectively. This observation originated from continuous Li dendrite propagation caused by repeated destruction and reconstruction of natural SEI layer. In addition, Z-COF@Cu exhibited a much higher response current and lower Li deposition overpotential (− 39 mV) compared to bare Cu (− 57 mV) in cyclic voltammetry (CV) curves at 0.5 mV s^−1^ (Fig. S8b, c), highlighting significant efficacy of Z-COF in facilitating Li^+^ transport. Highly reversible plating/stripping peaks at different scan rates from 0.1 to 0.5 mV s^−1^ (Fig. S8d) informed the excellent interfacial stability of Z-COF protective layer.

To assess the relationship between Z-COF protective layer and kinetic behaviors of Li plating/stripping process, a series of electrochemical tests was conducted using symmetrical cells. The exchange current density (*i*_0_) was obtained from the corresponding Tafel plots (Fig. 3c). The Z-COF@Li demonstrated a higher i_0_ value (0.55 mA cm^−2^) than that of bare Li (0.23 mA cm^−2^), illustrating a superior ion transport capability at the electrode interface modified with Z-COF. The fast kinetics migration characteristic at interfacial layer was further confirmed by Li^+^ transference number (t_Li_^+^). As shown in Fig. S9, Table S3, and Fig. 3d, the t_Li_^+^ improved remarkably from 0.42 for bare Li to 0.87 for Z-COF@Li. A higher t_Li_^+^ could effectively postpone the time of dendrite formation as governed by Sand’s model [48], which was beneficial to reduce interfacial polarization and promote even Li deposition. Meanwhile, the rate performances of symmetrical cells were examined at 2 mAh cm^−2^ with varied current densities (Fig. S10). When the current densities increased from 1 to 10 mA cm^−2^, the bare Li cells suffered an enormous raising in polarization voltage, revealing sluggish kinetics and a dendrite/dead Li-eroded LMAs surface. In stark contrast, Z-COF@Li had relatively constant cycling tendencies and displayed smaller voltage hysteresis throughout the entire process, further verifying its superior electrochemical reversibility and enhanced redox kinetic properties. Besides, temperature-dependent electrochemical impedance spectroscopy (EIS) on symmetrical cells was tested to further verify the critical function of Z-COF on the Li^+^ transfer. The EIS results in Fig. S11a, b manifested that the interfacial resistance of Z-COF@Li was lower than that of the counterpart bare Li anode at all the measured temperatures. Based on equivalent circuit model and fitting Nyquist data (Fig. S11c and Table S4), the activation energy (E_a_) for Li deposition was determined through Arrhenius equation [49]. The E_a_ determined to be 43.37 and 51.51 kJ mol^−1^ for Z-COF@Li and bare Li, respectively (Fig. 3e). The obviously reduced EIS and E_a_ implied that Z-COF protective layer was favorable for accelerating Li^+^ transmission, which was consistent with the low Li^+^ migration energy barrier in Fig. 1b. To quantitatively evaluate the Li^+^ conducting capability of Z-COF, ionic conductivity measurement was taken on the COF-based stainless steel symmetrical cells (SS|COF@LiTFSI-based electrolyte (COF@LE)|SS). As shown in Fig. S12, Z-COF exhibited the best ionic conductivity (*σ*_303K_ = 3.75 mS cm^−1^) and lowest Ea, compared to the anionic BDSA-COF and cationic EB-COF, suggesting the improved Li^+^ transport behaviors. These results confirmed the previous assertion that Z-COF possessed the low Li^+^ migration energy barrier.

Furthermore, the local chemical circumstance of Z-COF permeated in LiTFSI electrolyte was investigated by ^7^Li solid-state NMR spectroscopy. As illustrated in Fig. 3f, pure LiTFSI possessed a highly shielded environment for Li, while chemical shift of Z-COF@LiTFSI changed from − 1.19 to 0.13 ppm. The downfield shift indicated the interaction between Li^+^ and TFSI^−^ was weakened. It was ascribed to the attraction of negative sulfonate groups to Li^+^ and the anchoring of positive ethidium groups to TFSI^−^, which reduced electron cloud density surrounding the Li^+^ nucleus, promoted the dissociation of LiTFSI and finally enhanced mobility of Li^+^. To further elucidate this behavior, the molecular electrostatic potential (ESP) was calculated. As shown in Fig. 3g, the distribution of positively and negatively charged clusters was spatially isolated, indicating that the Z-COF held monodispersed zwitterionic units. Given the high polarizability of LiTFSI, the Z-COF with differential ESP distribution could provide two distinct polar adsorption sites for the dissociation of LiTFSI. Specifically, the strongest electron density (− 6.353 eV) was located around the sulfonate groups, indicating a special affinity for Li^+^ in this region. The highest ESP value (+ 3.180 eV) was observed for the ethidium groups, which possessed positively charged nitrogen ions and played a vital role in trapping TFSI^−^ anions of liquid electrolytes via electrostatic attraction. Overall, the integration of experimental and theoretical calculation collectively proved that the repetitive locally charged bulks in Z-COF protective layer could function as excellent dissociation enhancer for LiTFSI.

The effect of Z-COF protective layer on long-term cycling stability of LMAs was probed by galvanostatic charging/discharging test of symmetrical cells. As depicted in Fig. 3h, the cells with or without the Z-COF protection were cycled at 2 mA cm^−2^ /2 mAh cm^−2^. Bare Li electrodes only cycled for 370 h before suffering an enormous increase in overpotential. Conversely, the Z-COF@Li electrodes could steadily cycle for over 6300 h with no noticeable voltage fluctuation. Similar improvement was observed in the case that the current condition of 5mA cm^−2^/5 mAh cm^−2^ (Fig. 3i) and the Z-COF@Li symmetrical cells still achieved an extended cycle life up to 5200 h with a relatively lower overpotential, whereas bare Li exhibited continuous and substantial voltage fluctuation after 70 h. The improved performance could be stem from the following key factors. First, the orderly arranged nanochannels of Z-COF redistributed Li^+^ flux and induced homogeneous Li deposition. Second, sulfonate groups provided abundant hopping sites for Li^+^ transport. These beneficial effects synergistically stabilized the interface of LMAs and actuated rapid Li^+^ migration, ultimately leading to a pronounced enhancement in electrochemical performance (Fig. 3j).

**Fig. 4:**
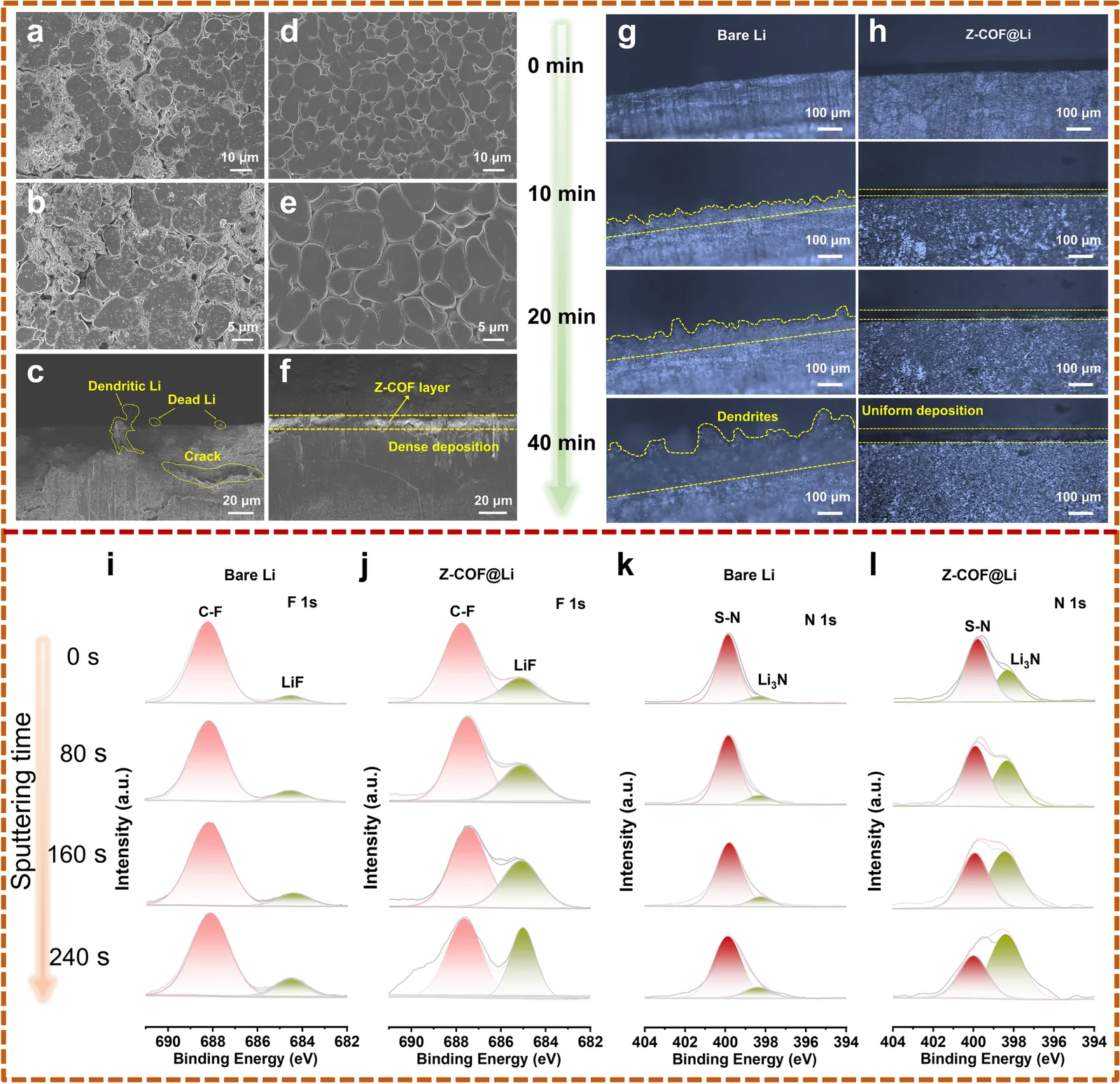
Surface and cross-sectional morphology of **a**–**c** bare Li and **d**–**f** Z-COF@Li anodes in Li|Li symmetrical cells after 200 cycles at 1 mA cm^−2^/1 mAh cm^−2^. In situ optical microscopy images of Li deposition on **g** bare Li and **h** Z-COF@Li at 2 mA cm^−2^. **i**–**l** XPS depth profiles of F 1*s* an N 1*s* spectra in Li metal anodes with and without Z-COF protective layer after 100 cycles, respectively

To visually clarify the ability of Z-COF protective layer to optimize Li deposition, the surface morphologies of LMAs were characterized by SEM. Compared with bare Li foil (Fig. S13a, b), a homogeneous 15-μm-thick Z-COF layer was evenly coated on the Li foil surface (Fig. S13c, d). As depicted in Fig. 4a, b, amounts of needle-like Li dendrites scattered on the irregular bulk spherical particles, multitudinous loose porous structure and dead Li appeared on the bare Li metal surface after 200 cycles at 1 mA cm^−2^/1 mAh cm^−2^. Besides, the cross-section image in Fig. 4c indicated the presence of branch-like unreactive Li and huge cracks. The transfer process of Li^+^ across this discontinuous interface was kinetically sluggish. More seriously, large voids exposed more fresh lithium to electrolyte, triggering severe parasitic reaction that pushed the breakdown of SEI layers and leaded to swift electrochemical properties deterioration. In comparison, a dense, smooth and dendrite-free Li deposition morphology was observed with introduction of Z-COF protective layer (Fig. 4d, e). The correspondingly cross-section image also displayed flat and compact Li plating layer (Fig. 4f). Collectively, these remarkable morphology differences informed that Z-COF could effectively suppressed the formation of Li dendrites, optimized the interfacial environment of Li metal/electrolyte and further ensured reversible Li deposition/dissolution. For distinctly observing the effect of Z-COF layer on inhibiting Li dendrite, in situ optical microscopy was conducted to dynamically inspect the evolution of Li deposition process at different time intervals at 2 mA cm^−2^. As described in Fig. 4g, Li dendrites were initially detected after plating 10 min on bare Li electrode. As the time extending, the mossy dendrites continued to grow, eventually developing into an unmanageable condition, which may trigger short circuit and trigger safety threats for LMBs. Conversely, the growth of dendrites was barely observed on the electrode modified with Z-COF throughout the working period (Fig. 4h).

XPS depth profiling was performed to determine the chemical composition of SEI formed on LMAs in Li|Li symmetrical cells after 100 cycles under 1 mA cm^−2^/1 mAh cm^−2^. As presented in F 1s and Li 1*s* spectra (Figs. 4i and S14), the peak intensity of LiF in Z-COF@Li electrode was much stronger than that of bare Li electrode as the sputtering time increasing. It indicated that the ion–dipole interaction between N^+^ in ethidium groups and F groups in TFSI^−^ was favorable for the breakage of C-F to generate a LiF-rich SEI layer. This result would be verified by DFT calculations later. The characteristics of high mechanical strength, excellent chemical stability and strong electron-insulation property endowed LiF as an optimal interfacial component in adjusting Li deposition and passivating electrode surface [50]. For N 1*s* spectra, the content of Li_3_N in bare Li did not undergo obvious change as the sputtering depth increased (Fig. 4j). Differently, the content of Li_3_N in Z-COF@Li exhibited upward tendency from surface to bulk (Fig. 4k), suggesting that the formation of Li_3_N-dominated inner layer SEI induced by Z-COF. In addition, the content of Li_3_N in Z-COF@Li was consistently higher than that in bare Li at the same sputtering time. The possible reason for this phenomenon was that the electrostatic attraction between N^−^ in TFSI^−^ and N^+^ in ethidium groups induced the generation of Li_3_N-rich SEI layer. The high ionic conductivity property of Li_3_N could reduce Li^+^ migration energy barrier and accelerate Li^+^ interfacial transport. Hence, based on XPS analysis, the LiF/Li_3_N-rich SEI layer at Z-COF@Li electrode made great contribution to enhance the interfacial stability and boost Li^+^ transport kinetics.

### Mechanistic Investigations of Z-COF Modulating Li^+^ Transport Behaviors

**Fig. 5:**
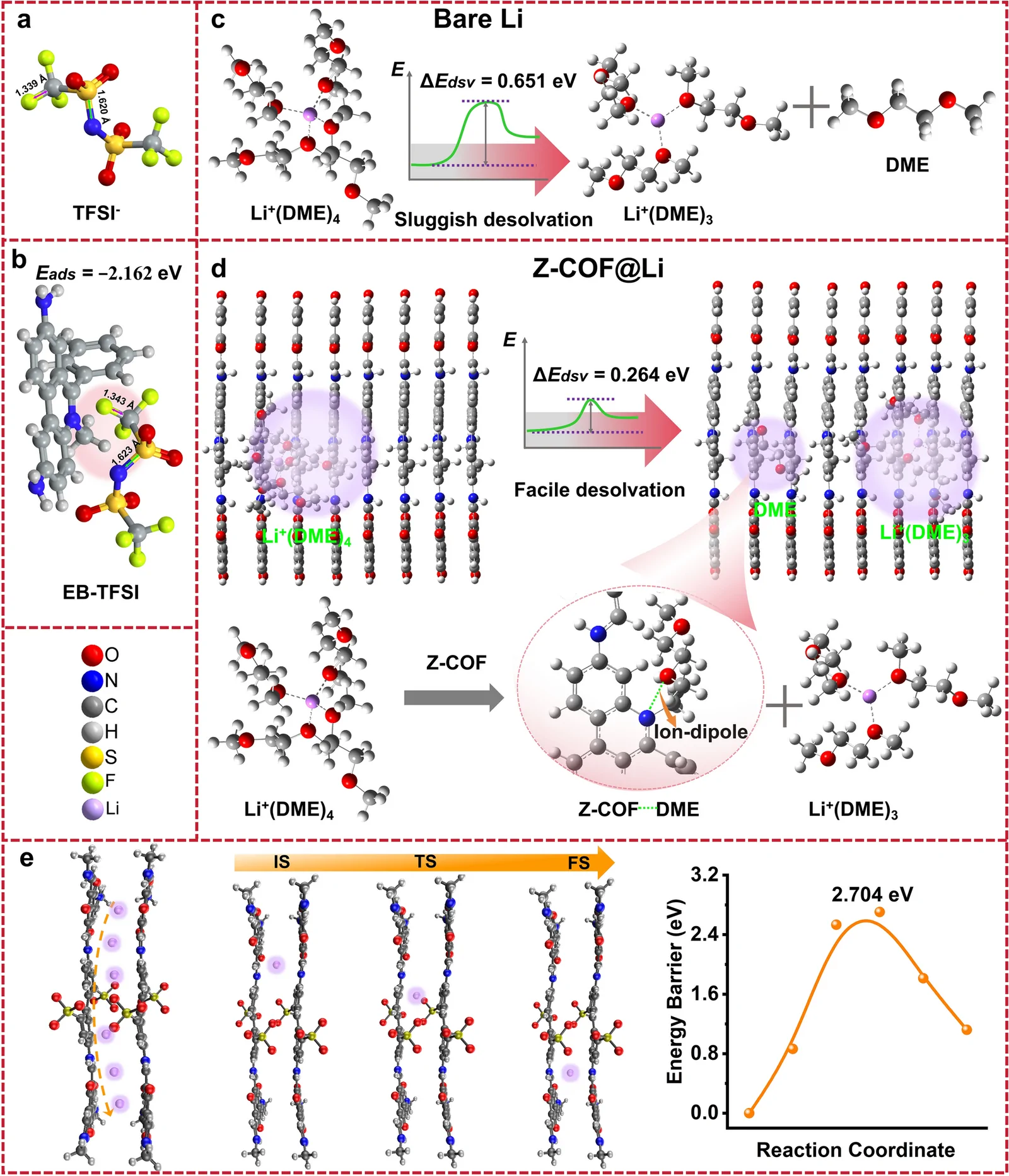
Density functional theory calculation. **a** Bond length of C-F and N-S in TFSI^−^. **b** Bond length of C–F and N–S in Z-COF-TFSI and the chemical coordination circumstance of simulated adsorption energy between TFSI^−^ and Z-COF with the terminal optimized geometries (the ethidium unit was selected as molecular fragment for DFT calculations). The desolvation energy of Li^+^(DME)_4_ to Li^+^(DME)_3_ + DME in electrolyte **c** with and **d** without Z-COF protective layer, respectively. **e** Theoretical illustration of Li^+^ migration pathway along the planar orientation inside Z-COF and the corresponding Li^+^ migration energy barriers. The initial, transition and final states were abbreviated as IS, TS and FS, respectively

The capture effect of ethidium groups in Z-COF on TFSI^−^ was validated by DFT calculation. As shown in Figs. 5a, b and S15 and Table S5, the substantial adsorption energy (− 2.162 eV) of TFSI-Z-COF indicated that the electron-deficient ethidium groups in Z-COF as anion capturer could attract the electron-rich TFSI^−^ and further promote interfacial charge transfer, thereby accelerating the decomposition of TFSI^−^. The bond length changes provided direct structural evidence corroborating the above conclusion. The C-F and S–N bond lengths in TFSI^−^ captured by ethidium sites were 1.343 and 1.623 Å, respectively, which both longer than those in traditional TFSI^−^ (1.339 and 1.620 Å). The increased bond length signified the C–F and S–N chemical bonds in trapped TFSI^−^ were more susceptible to cleavage, enabling the formation of LiF/Li_3_N-rich interphase.

According to prior research, the Li^+^ desolvation was recognized as a kinetically limiting step in Li^+^ transport at the electrode/electrolyte interface [51]. In the Li^+^ solvation sheath coordination structure of ether-based electrolytes, Li^+^ predominantly bounded to the C-O groups of dimethoxyethane (DME) and was solvated by four DME molecules at the first solvation shell to form a typical Li^+^(DME)_4_ cluster. Considering the exceptionally high polarity of DME [52], thus, the Li^+^(DME)_4_ was selected for simulating the desolvation process. The desolvation energy of Li^+^(DME)_4_ → Li^+^(DME)_3_ + DME was estimated using DFT calculation. As could be seen in Fig. 5c, d, the desolvation energy of Li^+^(DME)_4_ to Li^+^(DME)_3_ and DME with the introduction of Z-COF protective layer was 0.264 eV, much lower than that of the bare Li (0.651 eV), suggesting that zwitterions could act as “desolvation promoters,” weakened the interaction of Li^+^-solvent coordination, expedited the liberation of free Li^+^. The higher ratio of free DME/fixed DME with the addition of Z-COF (Fig. S16) indicated the establishment of ion–dipole interaction between DME and ethidium cations. The concerted application of positively charged ethidium sites anchoring DME molecules via ion–dipole interaction and negatively charged sulfonate sites concentrating Li^+^ via electrostatic attraction accelerated the facile and effective stripping of Li^+^ from solvent clusters. Furthermore, upon the dissociation of one DME molecule from the solvent clusters, the residual partially desolvated species preferentially facilitated the reduction of Li⁺ over the decomposition of solvent molecules [53]. Therefore, Z-COF simultaneously promoted Li⁺ desolvation and passivated interfacial side reactions.

The Li^+^ diffusion behaviors in nanochannels were investigated by comprehending the migration energy barrier along the planar and axial pathways of Z-COF pore. As illustrated in Figs. 1b and 5e, both axial and planar transfer pathways and their corresponding energy profiles within the Z-COF were calculated to elucidate the actual situation of Li^+^ migration. The energy barrier for Li^+^ migration along the axial orientation (0.809 eV) was lower than in the planar direction (2.704 eV), revealing that the intrinsic pore structure of Z-COF played a crucial role in governing Li^+^ transport. The possible reasons of Li^+^ migration along the axial pathway were as follows: (i) The ordered pore distribution and suitable pore diameter (17 Å) of Z-COF provided an optimal migration condition for Li^+^ of small size (0.76 Å); (ii) the lithiophilic sulfonate groups enriched Li^+^ within the nanochannels while repelling anions established an exclusive Li^+^ migration channel, which facilitated Li^+^ sieving and boosted Li^+^ transportation along the perpendicular pathway.

### Electrochemical Performance of Z-COF@Li|LFP Full Cells

**Fig. 6:**
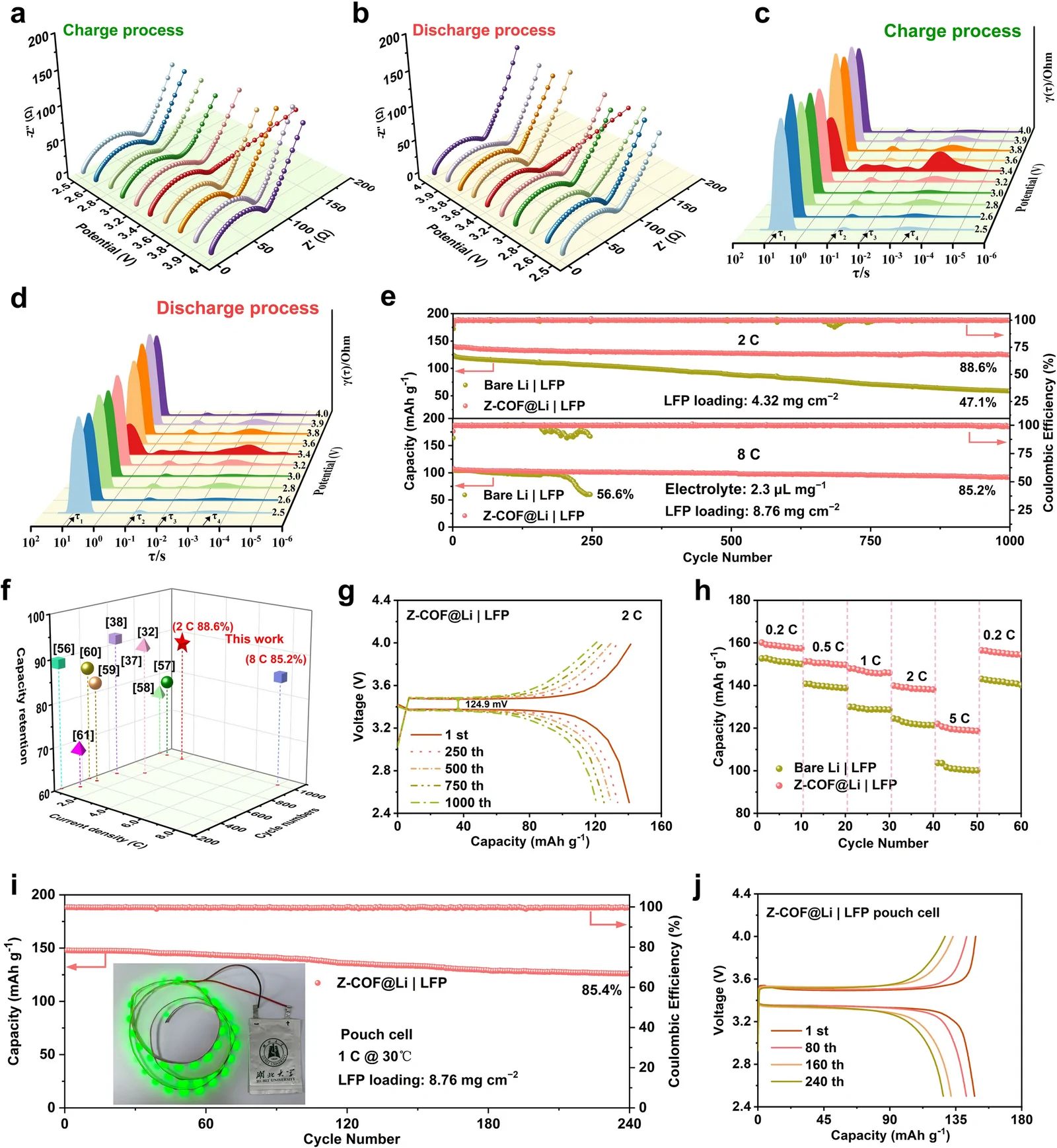
Electrochemical performances of full cells. **a** and **b** Three-dimensional images of the in situ EIS of Li|LFP full cells during the first discharge and charge process, respectively. **c** and **d** DRT calculated from the in situ EIS for the discharge and charge process, respectively. **e** Long-term cycling performance of the full cells with and without Z-COF protective layer at mild (2 C) and harsh condition (LFP loading: 8.76 mg cm^−2^, electrolyte amount: 2.3 μL mg^−1^, rate: 8 C). **f** Cycle performance comparison of Li|LFP full cells. **g** Galvanostatic charge–discharge curves of Li|LFP full cells with different cycles at 2 C. **h** Rate performance. **i** Long-term cycle stability and **j** corresponding specific capacity–voltage curves of Z-COF@Li|LFP pouch cell at 1 C

The interfacial impedance had a significant impact on modulating Li^+^ flow. Elevated impedance could diminish ionic/electronic conductivity, leading to the Li metal interfacial accumulation. This, in turn, promoted the generation of dendrites and dead Li. The reaction kinetics evolution during the continuous charge/discharge cycling was systematically examined through in situ EIS monitoring on the Li|LFP full cells. The changes in overall impedance at initial cycling stage are illustrated in Fig. 6a, b. Throughout the entire cycling process, the charge transfer impedance (*R*_ct_) values exhibited no significant changes in the high-frequency region, confirming that the establishment of a stable electrode interface ensured the steady working of Z-COF@Li|LFP full cell. According to the previous research reports, the total impedance spectrum was mainly contributed by multiple parts [54]. To provide a more accurate and intuitive assessment of the impedance contribution of each component, the distribution of relaxation time (DRT) method was applied to obtain a precise description of the charging/discharging processes during operation [55]. As shown in Fig. 6c, d, electrochemical signal feedback yielded different intensities at a specific time constant; each local maxima represented a resistance contribution to the overall polarization resistance. The high-frequency part (*τ* < 10^−4^ s) was ascribed to the resistance of the unchanged electrolyte bulk phase irrespective of the state of charge for the cell. The distributions between 10^−4^ and 10^−3^ s (*τ*_4_) reflected the interfacial resistance between the electrolyte and the LFP cathode. The signals distributed around 10^−1^–10^0^ s (*τ*_2_, *τ*_3_) were attributed to the interface resistance between the electrolyte and the LMAs. Furthermore, the distributions near 10^1^ s (*τ*_1_) denoted the resistance of Li^+^ diffusion. The polarization impedance (*τ*_1_, *τ*_2_, and *τ*_3_) displayed fewer variations during the entire charge/discharge process, certifying that the introduction of Z-COF protective layer improved cycling reversibility and interfacial stability of Li anode. However, at the characteristic plateau voltage of ~ 3.4 V, the *τ*_4_ exhibited markedly distinct compared to other voltage regions, which associated with the change of Li^+^ diffusion pattern. Specifically, the solid phase diffusion of Li^+^ intercalation/deintercalation replaced the liquid phase diffusion in electrolytes, which mitigated the diffusion rate to some degree.

The application potential of Z-COF@Li anode in practical batteries was evaluated by assembling the full cells with commercial LFP and LiNi_0.8_Co_0.1_Mn_0.1_O_2_ (NCM811) cathodes. As shown in Fig. 6e, Z-COF@Li|LFP full cell deliverd a relatively high initial capacity of 140.5 mAh g^−1^ at 2 C (LFP loading: 4.32 mg cm^−2^), which was higher than 124.3 mAh g^−1^ of bare Li|LFP. After 1000 cycles, Z-COF@Li|LFP cell maintained an outstanding capacity retention ratio of 88.6% with an average CE of 99.78%, extremely superior to bare Li|LFP. Moreover, the electrochemical performance of the Z-COF@Li|LFP full cells was also tested at the harsh condition (high LFP mass loading: 8.76 mg cm^−2^, lean electrolyte: 2.3 μL mg^−1^). Although the bare Li|LFP cells displayed a similar capacity to that of Z-COF@Li|LFP cells during the initial cycles when the current density increased to 8 C, it suffered an enormous decline after 190 cycles due to uncontrolled dendrite growth and continuous electrolyte depletion, resulting in an inferior capacity retention ratio of 56.6% during 247 cycles. On the contrary, the Z-COF@Li LFP cells achieved an initial capacity of 108.1 mAh g^−1^ with a remarkable capacity retention of 85.2% for 1000 cycles. Compared to previously reported COF-based materials (Fig. 6f) in LMBs [32, 38, 56–61], and even to the zwitterionic COF materials (Table S6) [42, 44, 45, 62, 63], Z-COF demonstrated significant advantages. Besides, the voltage hysteresis (VH) values of bare Li|LFP cells severely deteriorated to 538.9 mV (Fig. S17a, b). Strikingly different, the Z-COF@Li|LFP full cells slowly increased to 124.9 mV during 1000 cycles (Fig. 6g). Similarly, the VH with no significant change during 1000 cycles could be detected at 8 C (Fig. S18). Low VH suggested the stabilized Li anode surface and fast Li^+^ migration pathway enabled by the Z-COF protective layer decreased interfacial charge transfer resistance and improved Li^+^ transport kinetics during Li plating/stripping process. In addition, the Z-COF@Li|LFP cell revealed superior specific capacities of 160.1, 151.3, 148.0, 139.9, and 120.7 mAh g^−1^ at 0.2, 0.5, 1, 2, and 5 C (Figs. 6h and S17c, d), respectively, which was obviously better than those of bare Li|LFP cell (151.3, 139.7, 129.3, 122.5, and 100.4 mAh g^−1^, respectively). Notably, the Z-COF@Li|LFP cell still retained the high capacity of 156.5 mAh g^−1^ upon reverting to current density of 0.2 C, indicating the excellent cycle reversibility. More importantly, Z-COF@Li|LFP pouch cell (45 mm × 45 mm in size) was constructed to further explore the practical applicability of Z-COF@Li anode. Encouragingly, the Z-COF@Li|LFP pouch cell (8.76 mg cm^−2^) could stably operate 240 cycles with capacity retention of 85.5% (initial specific capacity: 147.8 mAh g^−1^) at 1 C (Fig. 6i). The cycling stability of pouch cell was further reflected in the specific capacity/voltage curves (Fig. 6j); the VH of Z-COF@Li|LFP pouch cell maintained stable during the cycling process without an obvious raise, indicating the high interfacial stability. To further demonstrate the general applicability of zwitterionic COF in high-energy–density LMBs, the full cells paired with commercial NCM811 cathodes were assembled. As shown in Fig. S19, the Z-COF@Li|NCM811 cells displayed a high initial discharge capacity of 183.9 mAh g^−1^ and a remarkable capacity retention of 88.2% after 200 cycles at 2 C, superior to that of bare Li|NCM811 cells. These findings confirm the feasibility of employing Z-COF as an artificial protective layer for practical high-energy-density LMBs.

## Conclusion

In summary, a zwitterionic COF (Z-COF) was designed for regulating Li^+^ transport and stabilizing LMAs. On the one hand, the framework incorporating both cationic and anionic sites modulated the interface charge distribution, restraining Li dendrite growth originated from concentration polarization. On the other hand, the highly electronegative and lithiophilic sulfonate groups combined with ordered micropores in Z-COF fabricated exclusive Li^+^ migration channels, kinetically facilitating Li^+^ diffusion. Moreover, the local zwitterionic groups accelerated the dissociation of LiTFSI and the capture of TFSI^−^, promoting the breakage of C–F and S–N bonds and inducing the formation of LiF/Li_3_N-rich SEI. Finally, the ion–dipole interactions between ethidium cations and solvent molecules enhanced Li^+^ desolvation ability, thereby eliminating interfacial side reactions. The afore-mentioned advantages have been proved by the DFT calculation and detailed experimental characterizations. When served as artificial protective layer, Z-COF@Li symmetric cells delivered ultra-high Li^+^ transfer number of 0.87 and cycle lifespan exceeding 6300 h with low overpotential at the current density of 2 mA cm^−2^. The Li|Z-COF@Cu half cells delivered a high CE of 99.7% after 550 cycles. The Z-COF@Li|LFP full cells exhibited a high initial capacity of 108.1 mAh g^−1^ and outstanding capacity retention of 85.2% for 1000 cycles at 8 C even under the harsh conditions (LFP mass loading: 8.76 mg cm^−2^, electrolyte: 2.3 μL mg^−1^). More importantly, the assembled Z-COF@Li|LFP pouch cells demonstrated a lifespan of more than 240 cycles without obvious voltage fluctuation. The design concept of zwitterionic COF used as protective layer in boosting Li^+^ migration kinetic behaviors and stabilizing Li metal anodes illuminated the way toward the practical application of high-performance LMBs.

## Supplementary Information

Below is the link to the electronic supplementary material.

Supplementary file1 (DOCX 8462 KB)
