# Supplementary material for: Regulating Li+ Transport and Interfacial Stability with Zwitterionic COF Protective Layer Towards High-Performance Lithium Metal Batteries
Source: Nanomicro Lett. 2026 Jan 5;18:163. doi: 10.1007/s40820-025-02017-3 (PMC12765773; doi:10.1007/s40820-025-02017-3)
Supplement: Supplementary file 1 — Supplementary file1 (DOCX 8462 KB) [file 40820_2025_2017_MOESM1_ESM.docx]

Supporting Information for

**Regulating Li^+^ Transport and Interfacial Stability with Zwitterionic COF Protective Layer towards High-Performance Lithium Metal Batteries**

Liya Rong^1^, Yifeng Han^1^, Chi Zhang^1^, Hongling Yao^1^, Zhaojun He^1^, Xianbao Wang^1^, Zaiping Guo^1,2,^* and Tao Mei^1,^*

^1^ Hubei Collaborative Innovation Center for Advanced Organic Chemical Materials, Overseas Expertise Introduction Center for Discipline Innovation (D18025), Key Laboratory for the Green Preparation and Application of Functional Materials, Hubei Key Laboratory of Polymer Materials, College of New Energy and Electrical Engineering, Hubei University, Wuhan 430062, P. R. China

^2^ Department of Materials Science and Engineering, City University of Hong Kong, Kowloon, Hong Kong 999077, P. R. China

*Corresponding authors. E-mail: [meitao@hubu.edu.cn](mailto:meitao@hubu.edu.cn) (Tao Mei); [zaipiguo@cityu.edu.hk](mailto:zaipiguo@cityu.edu.hk) (Zaiping Guo)

S1 Materials

1,3,5-triformylphloroglucinol (TP), ethidium bromide (EB) and 4,4'-diaminobiphenyl-2,2'-disulfonic acid (BDSA), dioxane, mesitylene, 12 M acetic acid, N, N-dimethylformamide (DMF), Tetrahydrofuran (THF), ethanol and methanol were purchased from Aladdin Chemistry Co., Ltd. Liquid electrolyte of 1 M LiTFSI in DOL/DME (1:1) with 2% LiNO_3_ was purchased from DoDoChem Suzhou. All reagents in the experiment were chromatographically pure/analytical grade without further purification.

S2 Characterization

The morphologies and microstructures of the samples were investigated by using Field emission scanning microscopy (FESEM, Zeiss, Germany) equipped with electron dispersive X-ray spectroscopy (EDS) mappings and high-resolution transmission electron microscopy (HRTEM, FEI Tecnai G2 F20 X-Twin, USA). Powder X-ray diffraction (PXRD) was performed using a Bruker D8 Advance X-ray diffractometer at 40 kV and 40 mA with Cu Kα radiation, scanning from 2θ = 3 ° to 40 °. The X-ray photoelectron spectroscopy (XPS) characterization was conducted on a Thermo Escalab 250Xi spectrometer with monochromatic 150 W AlK(α) radiation. Fourier transform infrared (FT-IR) spectra of COF was identified by the Nicolet 6700 spectrometer (Thermo Fisher Scientific, U.S.A.). The specific surface area and pore size distribution were assessed by a Brunauer-Emmett-Teller (BET, ASAP 2460 surface area analyzer Micromeritics Co., Norcross, GA). Solid-state ^13^C cross-polarization magic-angle spinning NMR spectra (^13^C CP/MAS NMR) was taken in a Bruker Avance 600 M NMR spectrometer. Thermo gravimetric analyses (TGA) were carried out on a TG50 analyzer (Perkin Elmer Diamond TG/DTA, USA) within a temperature range of 25-800 °C in N2 with a heating rate of 10 °C/min. Elemental analyses (C, N, H) were tested via a Thermo Scientific Flash 2000 CHNS-O analyzer. Zeta potential was measured using Zerasizer nano ZSE in the conductivity range of 0-200 mS/cm. The dendrite growth process was observed by assembling an in-situ cell (LIB-MS, Beijing Scistar Technology Co. Ltd) equipped with quartz window on top and in-situ optical microscopy measurement (LW750LJT, CEWEI, Beijing).

S3 Cells assembly and test

The as-prepared electrodes were examined using CR2032 coin type cells. The cell assembly was conducted in the argon-filled glove box (Etelux Lab 2000) with H2O and O2 content level < 0.1 ppm and examined using LAND-CT3001A battery test system and electrochemical workstation (CHI660E, Chenhua).

**Li|Cu half cells:** Bare Cu or Z-COF@Cu was used as working electrode and the Li foil acted as counter electrode in Li|Cu half cells. A commercial Clegard 2400 polypropylene film as separator, and 60 μL of 1 M LiTFSI in 1,3-dioxolane (DOL) and 1,2-dimethoxyethane (DME) (volume ratio, 1:1) with 2wt% LiNO3 was used as electrolyte. For electrochemical measurements, the fresh Li|Cu half cells were activated for 3 cycles at 50 μA cm^−2^ between 0.1 and 1.0 V to purify surface and stabilize SEI. Then, the cells were plating/stripping at the current density of 0.5 mA cm^−2^ and areal capacity of 0.5 mA h cm^−2^, and the cut-off voltage was 1.5 V (vs. Li/Li^+^).

**Li|Li symmetrical cells:** Bare Li or Z-COF@Li was used as both sides of electrodes in symmetrical cells. A Celguard 2400 microporous film was regarded as separators and 80 μL of 1 M LiTFSI in DOL/DME (volume ratio of 1:1) with 2wt% LiNO3 was used as electrolyte. The Li|Li symmetrical cells were cycled at different current densities and deposition capacities: 2 mA cm^−2^/2 mA h cm^−2^ and 5 mA cm^−2^/5 mA h cm^−2^.

**Li|LFP full cells:** The as-prepared LFP electrodes were used as the cathode. The bare Li or Z-COF modified Li was used as the anode. Celguard 2400 microporous film was used as the separator. 1 M LiTFSI in DOL/DME (volume ratio of 1:1) with 2wt% LiNO_3_ was used as electrolyte. Galvanostatic charge-discharge tests were carried out with the potential range from 2.5 to 4.0 V (vs. Li/Li^+^) at different current densities. The Z-COF@Li |LFP pouch cell (length × width = 45 mm × 45 mm in size, LFP mass loading: 8.76 mg cm^−2^, amount of liquid electrolyte:12.3 μL mg^−1^) was fabricated by sealing with an Al pouch as a packaging substance. The entire fabrication process of the cells was performed in a dry room at room temperature. The long cycling test was conducted in a voltage window of 2.5-4.0 V (vs. Li/Li^+^) at 1 C (1 C = 170 mA g^−1^).

**Electrochemical impedance spectroscopy (EIS)**: The ex-situ EIS measurements were performed on electrochemical workstation in the frequency range from 0.1 Hz to 100 kHz with an alternating current amplitude of 5 mV at room temperature. In-situ impedance measurements data during constant-current charging and discharging of LFP full cells was synchronized acquisition. The voltage window was controlled in the range of 2.5-4.0 V. At each specific voltage, the cells were held for 5 minutes to ensure stabilization before measuring the impedance. Other test parameters are the same as those in the ex-situ impedance tests.

**Li^+^ transference number (𝑡Li^+^)**: The t_Li_^+^ was tested according to Bruce-Vincent-method by alternating current (AC) impedance and potentiostatic directcurrent (DC) polarization, which was calculated by using the following equation [S1]:

$$\text{t}_{\text{Li}^{\text{+}}}\text{=}\frac{\text{ Is(}\text{∆}\text{V - I}\text{0}\text{R}\text{0}\text{)}}{\text{I}\text{0}\text{(}\text{∆}\text{V - I}\text{S}\text{R}\text{S}\text{)}}\text{ (S1)}$$

where I0 and IS are the initial and steady-state current value, R0 and RS are the initial and steady-state interfacial resistances, and ΔV is the potential of potentiostatic DC polarization, respectively. t_Li+_ of Li/Li symmetric cells was measured with a voltage polarization of 10 mV for 1000 s at 25 °C.

**Tafel exchange current density**: Linear sweep voltammetry (LSV) for Li symmetric cells was conducted at a scanning rate of 0.2 mV s⁻¹ in a voltage window of −0.2–0.2 V to obtain the Tafel curves and calculated the corresponding exchange current densities (i_0_) to quantitatively describe the Li^+^ exchange value on the electrode surface according to the Bulter-Volmer equation [S2]:

$$\text{ln}\left( \text{i} \right)\text{= ln}\left( \text{i}_{\text{0}} \right)\text{+}\frac{\text{αnFη}}{\text{RT}}\text{ (}\text{S2}\text{)}$$

where α, n, F, η, R, and T represents the charge transfer coefficient, electron transfer number, Faraday constant, overpotential, universal gas constant, and absolute temperature, respectively, while i stands for the current density.

**Ion conductivity of COF@LiTFSI-based electrolyte (COF@LE):** The pure COF powders were immersed in the LiTFSI-based electrolyte (1 M LiTFSI dissolved into a mixture of DOL/DME, v/v=1/1) for 2 h for activation. Then, the samples were vacuum-dried over dust free paper and pressed into electrolyte pellets under gentle pressure. The thickness of the electrolyte membrane was ~ 250 μm. Finally, the COF@LE was tested for ionic conductivity in the blocking SS|COF@LE|SS symmetrical cell configuration via EIS. The Li^+^ conductivity (σ, S cm^–1^) was calculated according to the following equation:

$$\text{}\text{ }\text{= (L)/(RS)}\text{ }\text{ (}\text{S3}\text{)}$$

where *L* is the thickness of the solid pellet (cm), 𝑅 is the bulk resistance (Ω), and *S* is electrode area (cm^2^), respectively.

**Activation energy**: The activation energy was measured by the temperature-dependent EIS of Li|Li symmetrical cells. The activation energy (Ea) was calculated by the Arrhenius equation [S3]:

$$\text{ln}\left( \text{k} \right)\text{= }\text{-}\frac{\text{Ea}}{\text{RT}}\text{ }\text{+lnA}\text{ }\text{ (}\text{S4}\text{)}$$

where k, A, Ea, R, and T correspond to the rate of a reaction, Frequency factor, activation energy of the reaction, gas constant and absolute temperature (range from 303 to 343K), respectively.

**S4 Theoretical calculations**

The Density functional theory (DFT) calculations [S4, S5] were performed in the Gaussian 16 software package. The optimization of molecular geometric structure was conducted using B3LYP/6-31G(d) basis set [S6]. The adsorption energies (E_ads_) were calculated as E_ads_ = E_total_ - E_A_ - E_B_, where E_total_, E_A_, and E_B_ are the total energies of the optimized adsorbate/substrate system, the adsorbate in the structure, and the clean substrate, respectively. The electrostatic potential (ESP) maps and values were obtained using VMD and Multiwfn [S7, S8]. The desolvation energy of Li^+^(DME)_4_ (E_dsv_) in liquid electrolyte was calculated by the following equation: E_dsv_ = E_Li(DME)3+_ + E_DME_ – E_Li(DME)4+_, where E_Li(DME)3+_, E_DME_, and E_Li(DME)4+_ were the energies of Li(DME)_3_^+^, DME, and Li(DME)_4_^+^, respectively. E_dsv_ in the liquid electrolyte with a COF framework was calculated by the following equation: E_dsv_ = E_COF-Li(DME)3+_ + E_COF-DME_ – E_COF-Li(DME)4+_ – E_COF_, where E_COF-Li(DME)3+_, E_COF-DME_, E_COF-Li(DME)4+_ and E_COF_ were the energies of Li(DME)_3_^+^ with COF framework, DME with COF framework, Li(DME)_4_^+^ with COF framework, and bare COF framework, respectively [S9]. The migration pathway was determined via Intrinsic Reaction Coordinate (IRC) calculations at the B3LYP/6-31G(d) level. First, the transition state (TS) was located using the Berny algorithm and confirmed by a single imaginary frequency in the Hessian analysis. Then, forward and backward IRC calculations were performed to trace the minimum energy path (MEP) connecting the reactant and product states. The calculations were conducted in the Gaussian 16 program, and the pathway was visualized using IQmol package.

**Supplementary Figures and Tables**


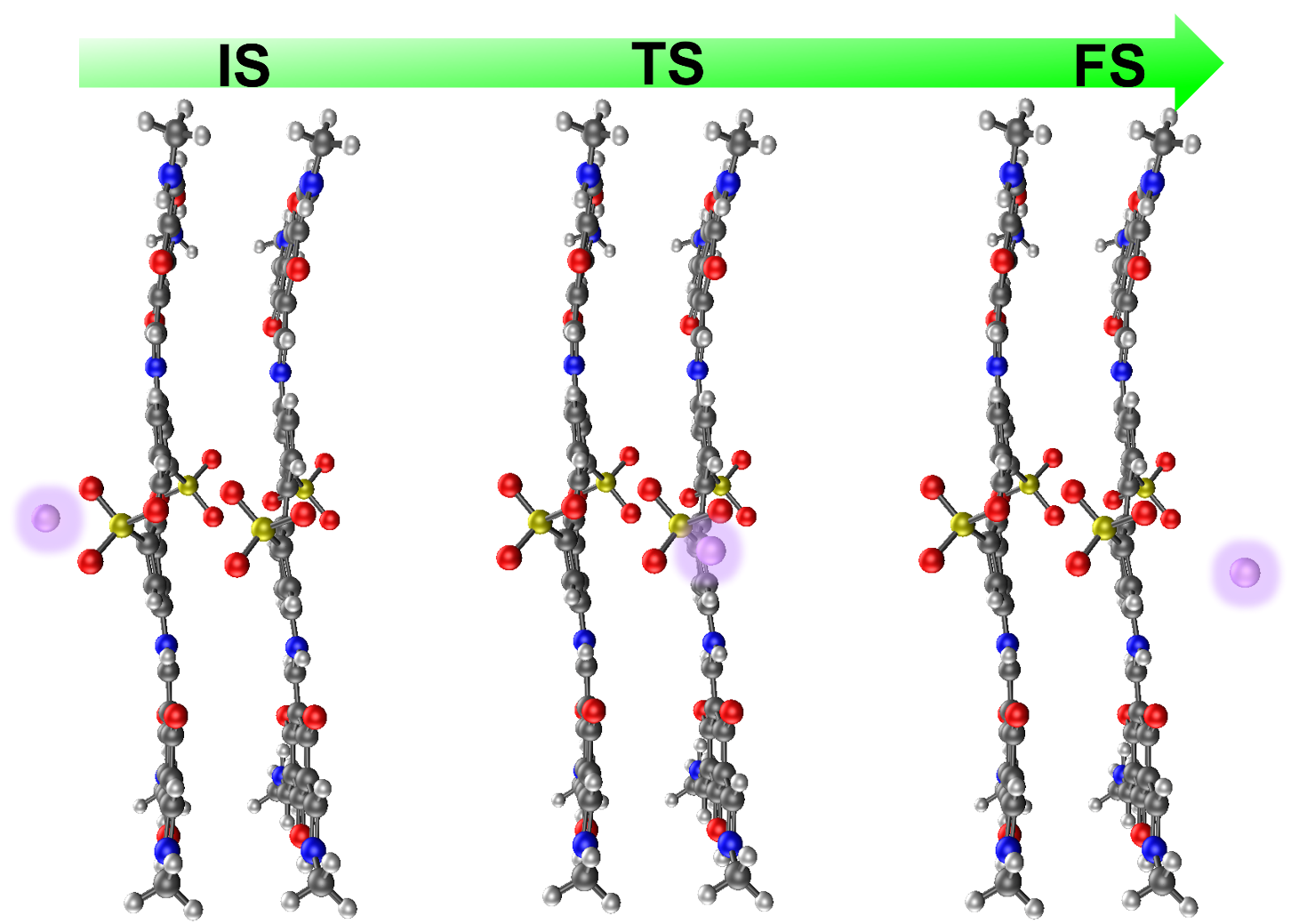


**Fig. S1** The initial (IS), transition (TS), and final (FS) states of the Li⁺ migration pathway along the axial orientation inside Z-COF


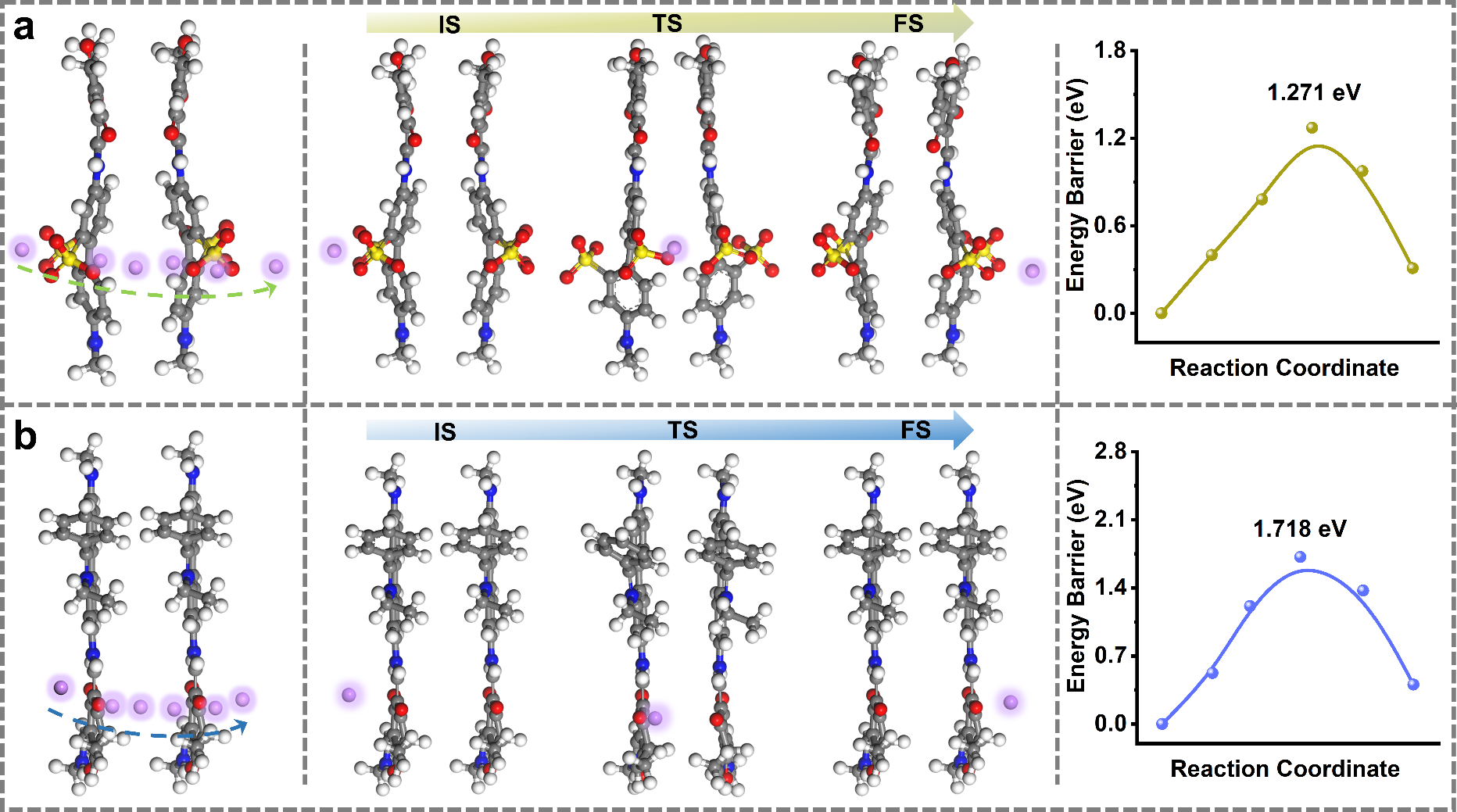


**Fig. S2** Theoretical illustration of Li^+^ migration pathway along the axial orientation inside the (**a**) anionic COF (BDSA-COF) and (**b**) cationic COF (EB-COF) with corresponding Li^+^ migration energy barriers. The initial, transition, and final states were abbreviated as IS, TS, and FS, respectively


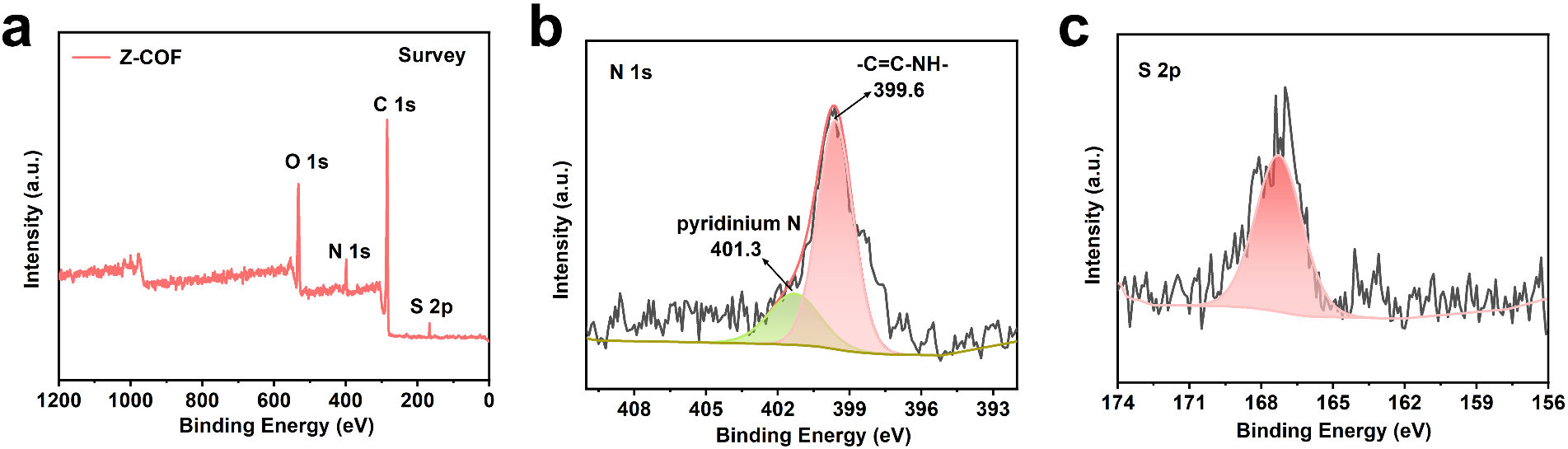


**Fig. S3** (**a**) High-resolution XPS spectra, and corresponding (**b**) N 1, (**c**) S 2p XPS spectra for Z-COF


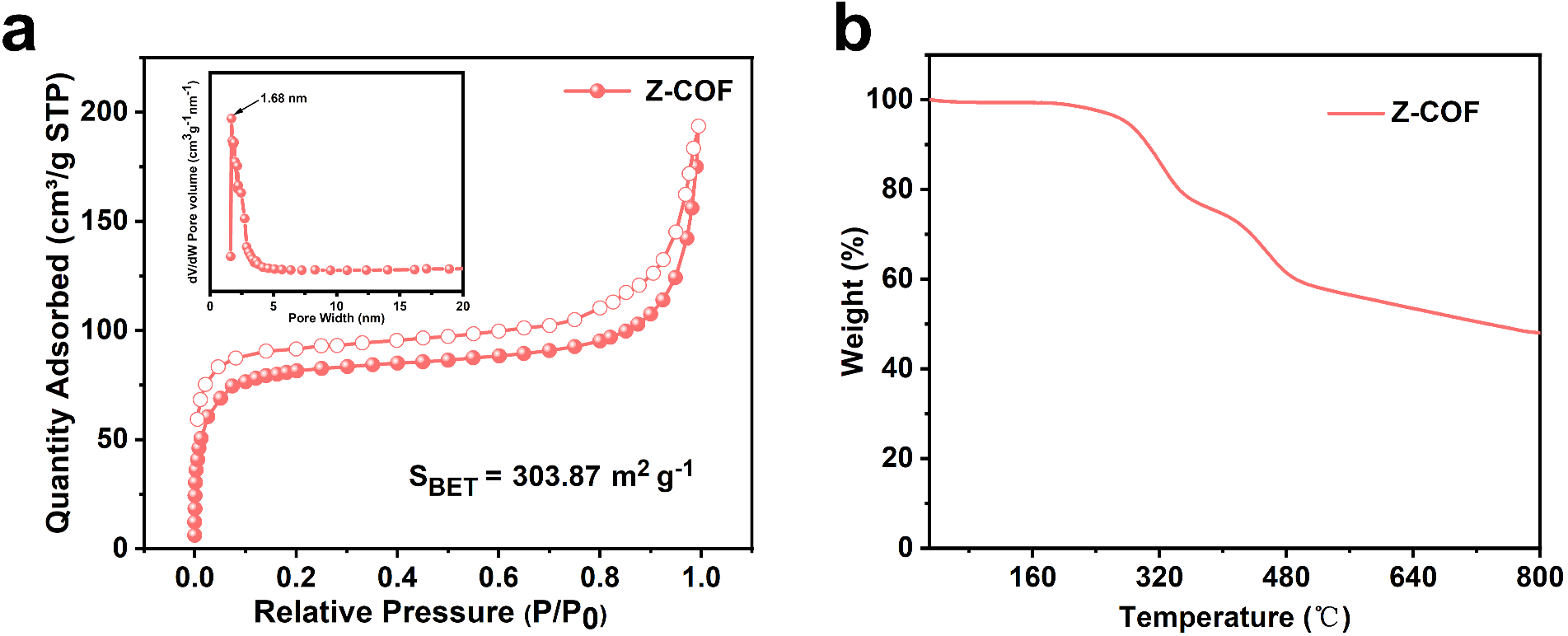


**Fig. S4** (**a**) Nitrogen absorption-desorption isotherms of Z-COF (Inset: the pore size distribution). (**b**) TGA of Z-COF in the nitrogen atmosphere


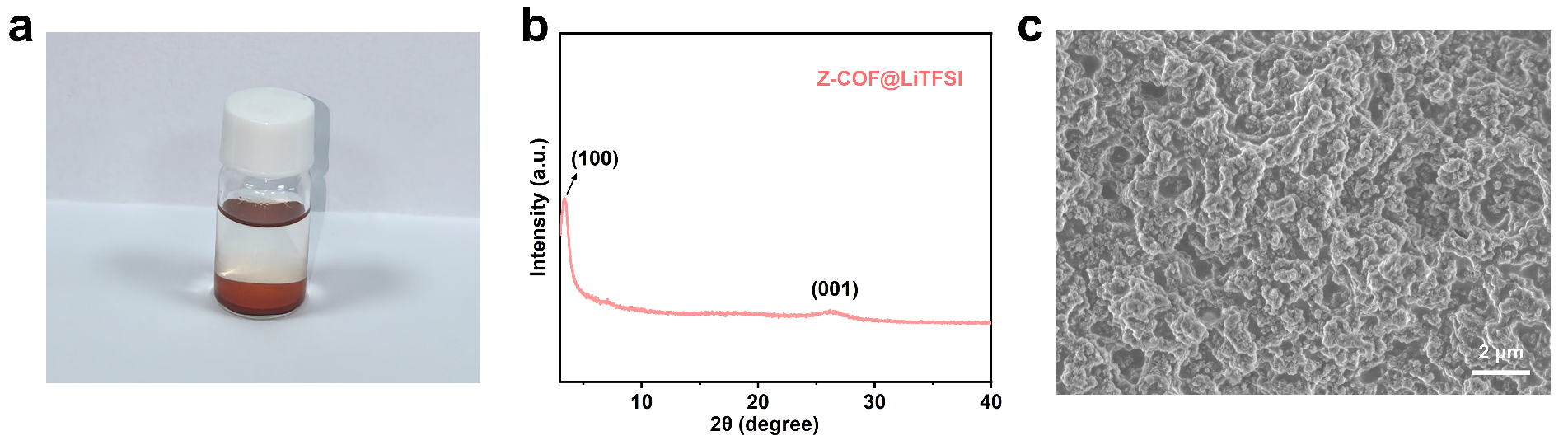


**Fig. S5** The optical photo (**a**) and PXRD pattern (**b**) of Z-COF immersed in TFSI-based electrolyte for three weeks. (**c**) SEM image of surface morphology for Z-COF@Li electrodes after 50 cycles at 1 mA cm^−2^ with a capacity of 1 mAh cm^−2^


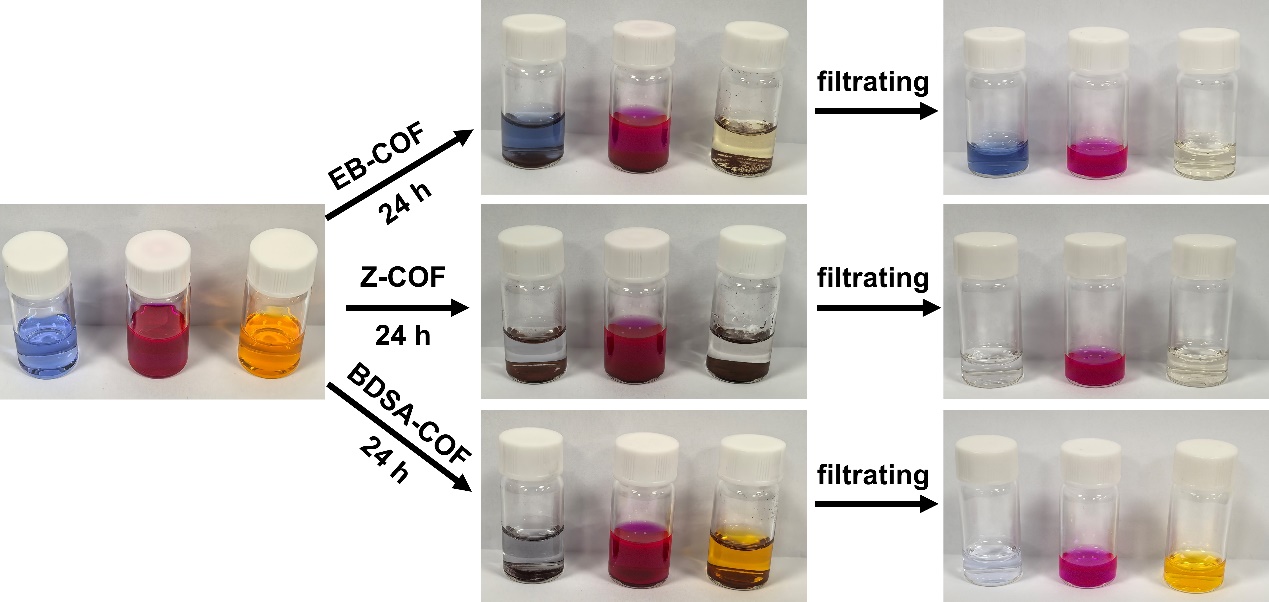


**Fig. S6** Dye adsorption experiment of Z-COF and its corresponding anionic COF (BDSA-COF) and cationic COF (EB-COF). From left to right, the dyes are methylene blue (positively charged), Nile red (neutral), and methyl orange (negatively charged), respectively. (note: the last set of pictures mean partial filtrate)


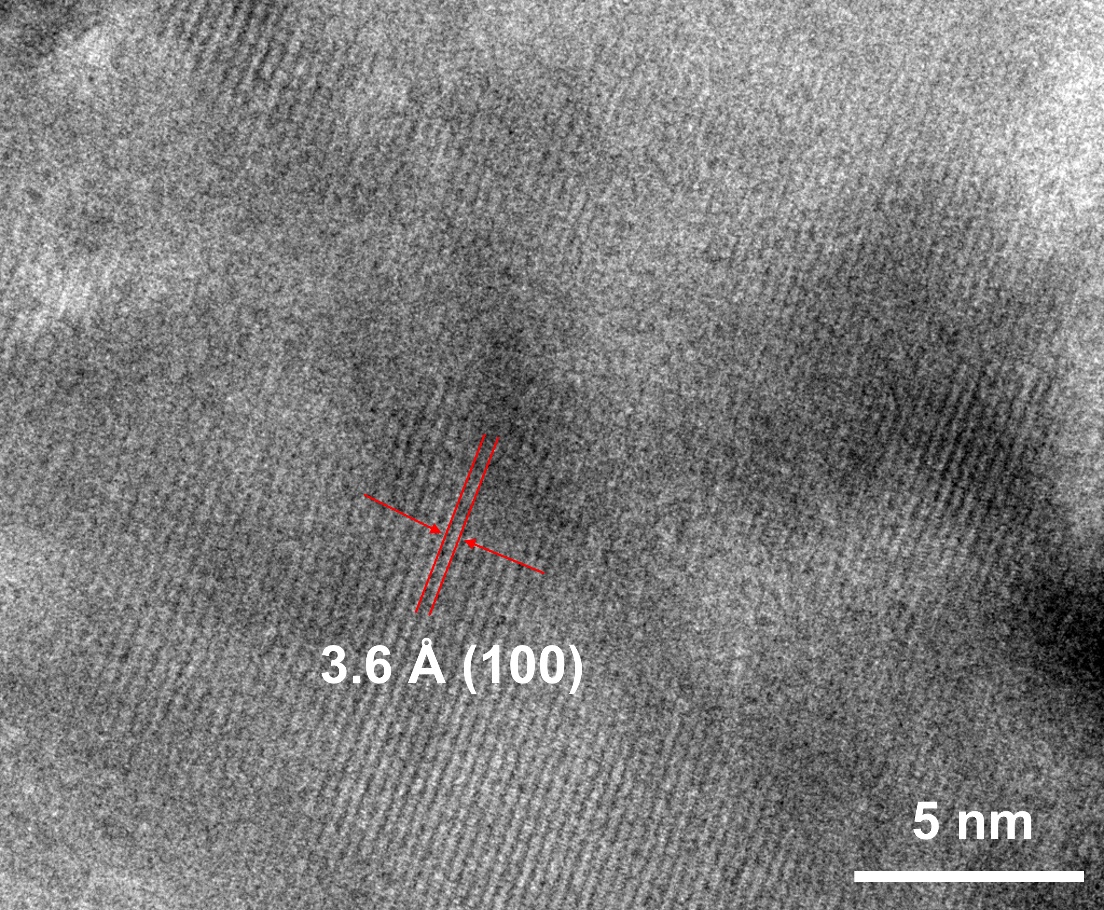


**Fig. S7** lattice fringes of Z-COF


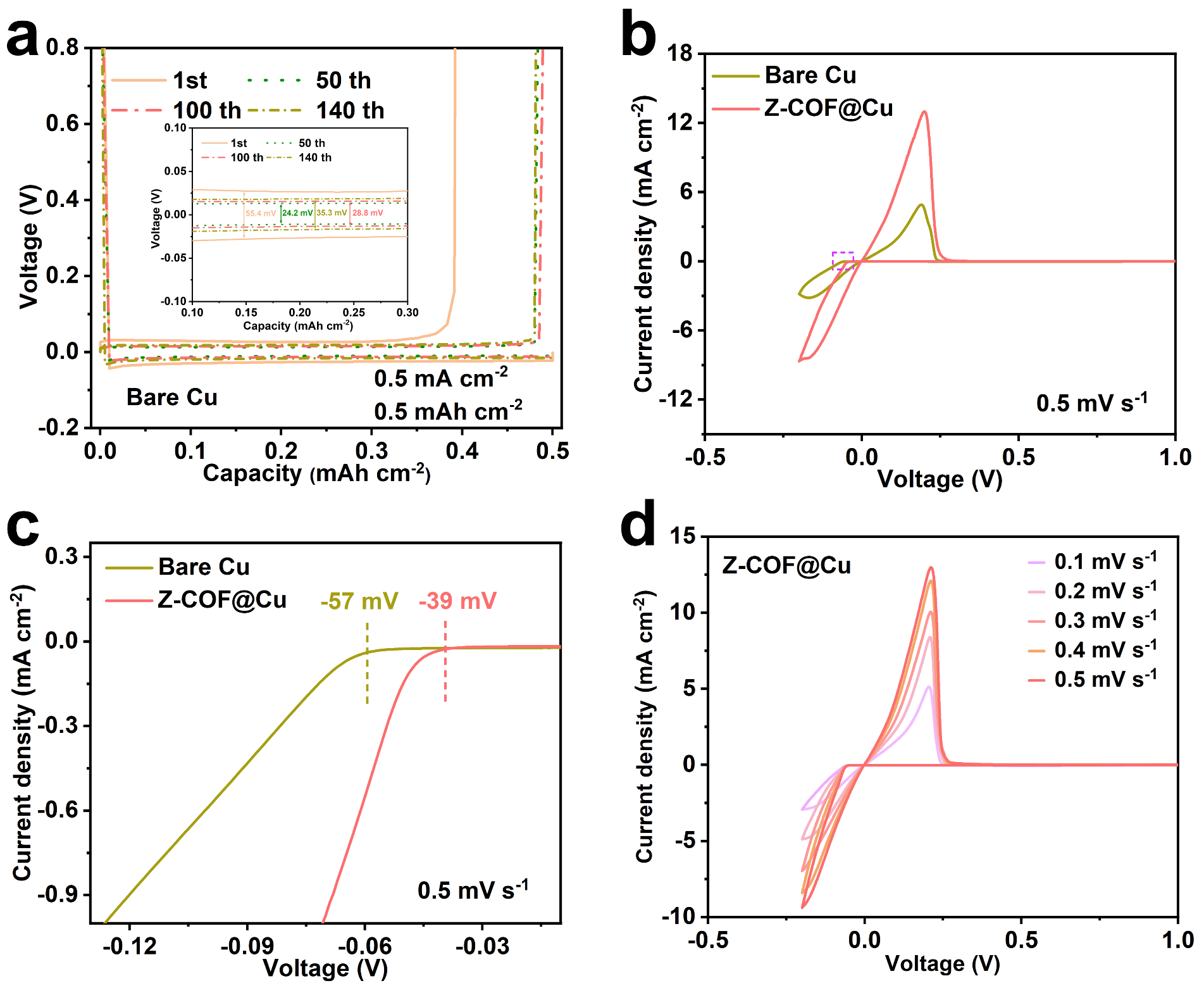


**Fig. S8** (**a**) The voltage-capacity curves of Li|bare Cu half cells, inset: partial enlarged detail. (**b**) Cyclic voltammetry curves of Li||Cu cells based on bare Cu and Z-COF@Cu at a scan rate of 0.5 mV s^−1^ from 1 to −0.2 V, and (**c**) corresponding partial enlargement. (**d**) CV curves from 0.1 to 0.5 mV s^−1^ of the Z-COF@Li|Cu half cells at different scan rates


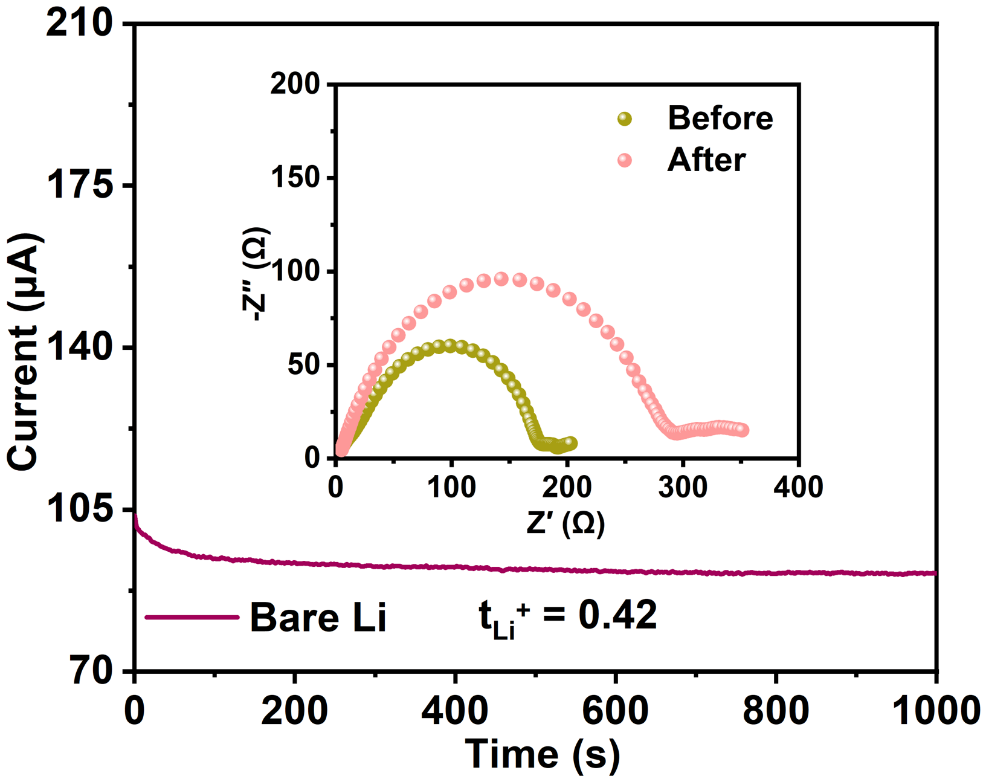


**Fig. S9** The Li^+^ transference number (t_Li_^+^) of bare Li symmetrical cell (inset: Nyquist plots of impedance before and after polarization)


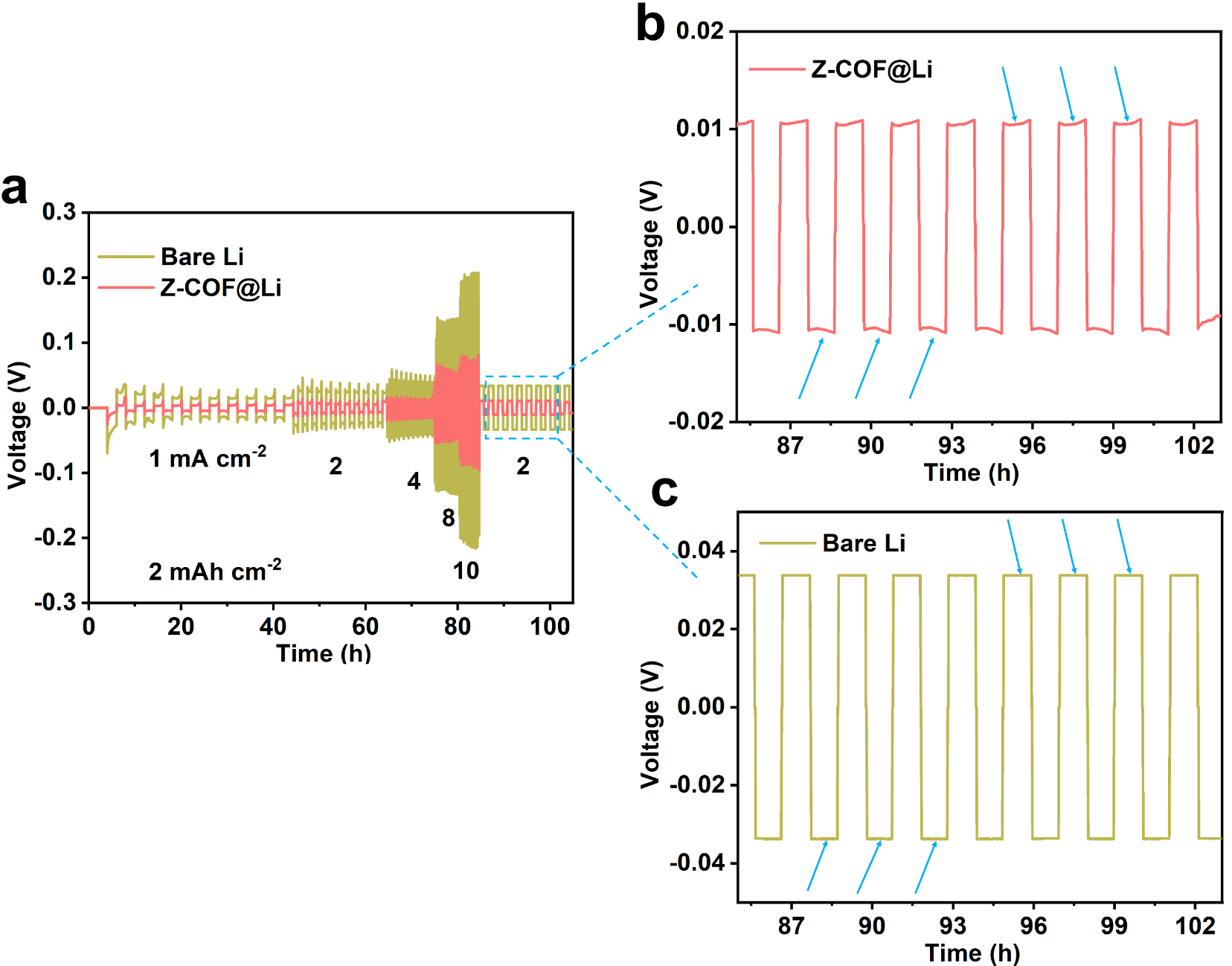


**Fig. S10** (**a**) The rate performance of Li|Li symmetrical cells assembled with different electrodes from 1 to 10 mA cm^−2^. The corresponding partial enlargement of (**b**) bare Li and (**c**) Z-COF@Li symmetrical cells at the second cycle of 2 mA cm^−2^, respectively


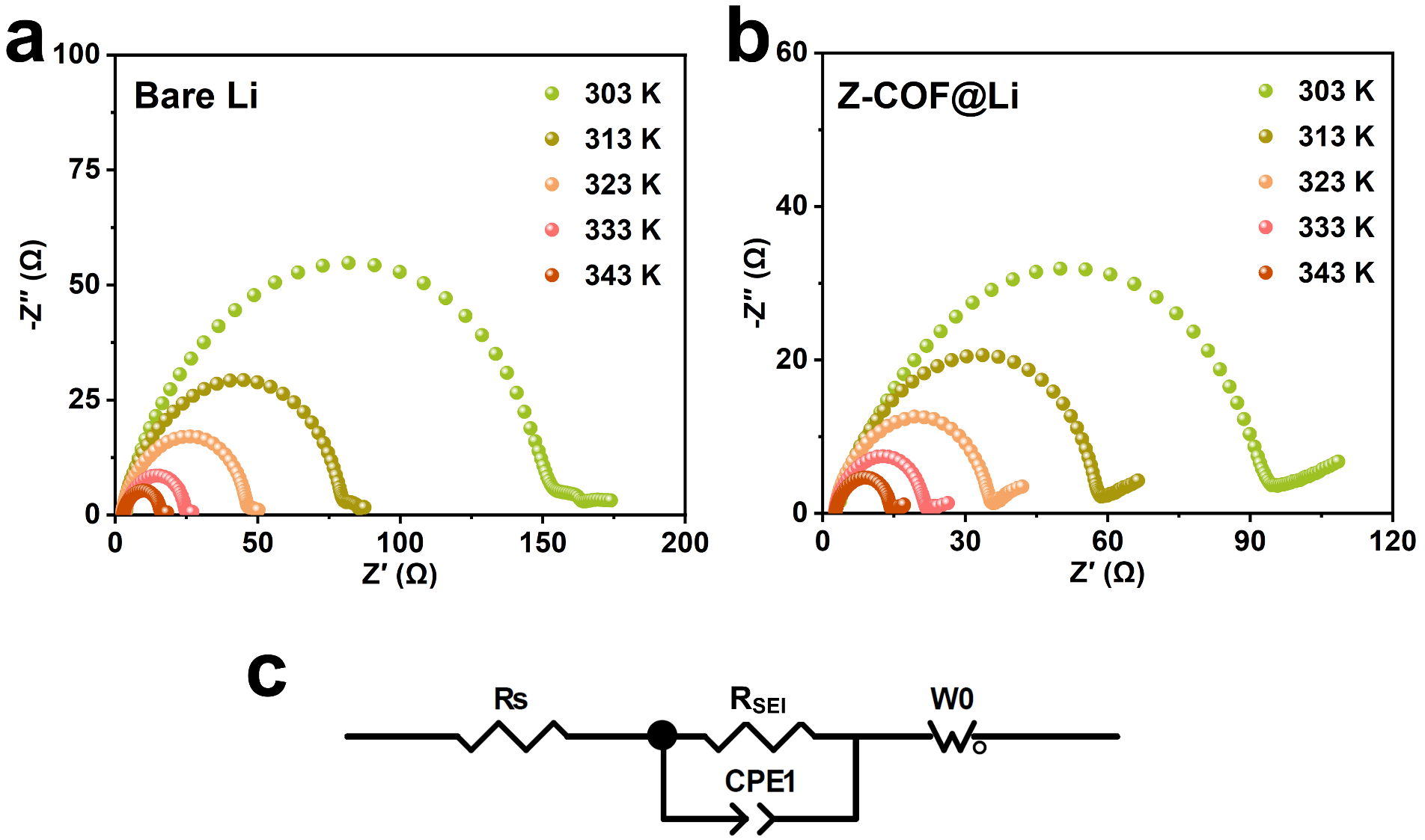


**Fig. S11** Nyquist plots of symmetric cells at different temperatures in (**a**) bare Li and (**b**) Z-COF@Li electrodes. (**c**) The equivalent circuit models for fitting Rs, RSEI.


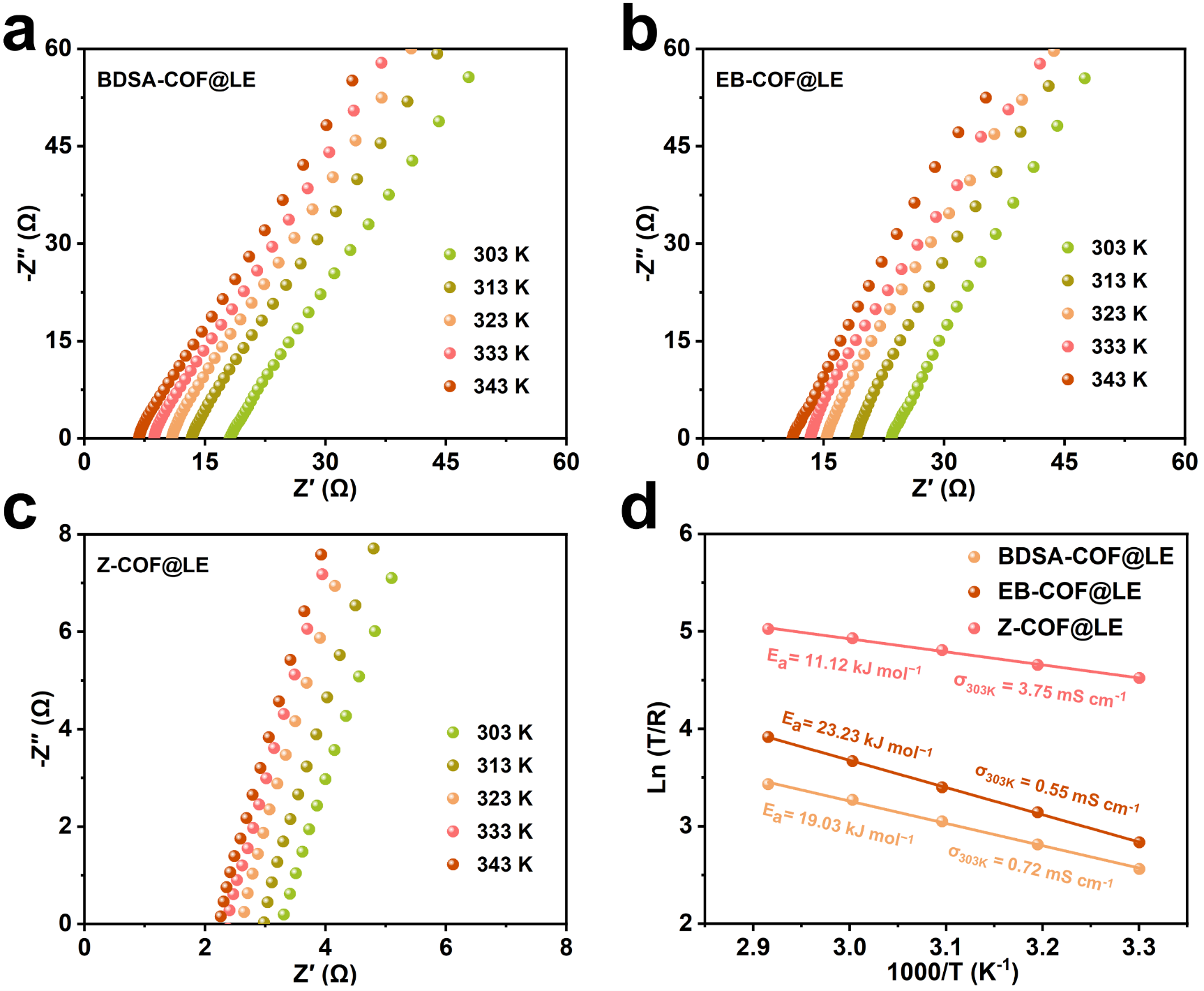


**Fig. S12** Nyquist plots of (**a**) BDSA-COF@LE, (**b**) EB-COF@LE and (**c**) Z-COF@LE at various temperatures. (**d**) Arrhenius plot of the ionic conductivities of BDSA-COF@LE, EB-COF@LE and Z-COF@LE at various temperatures


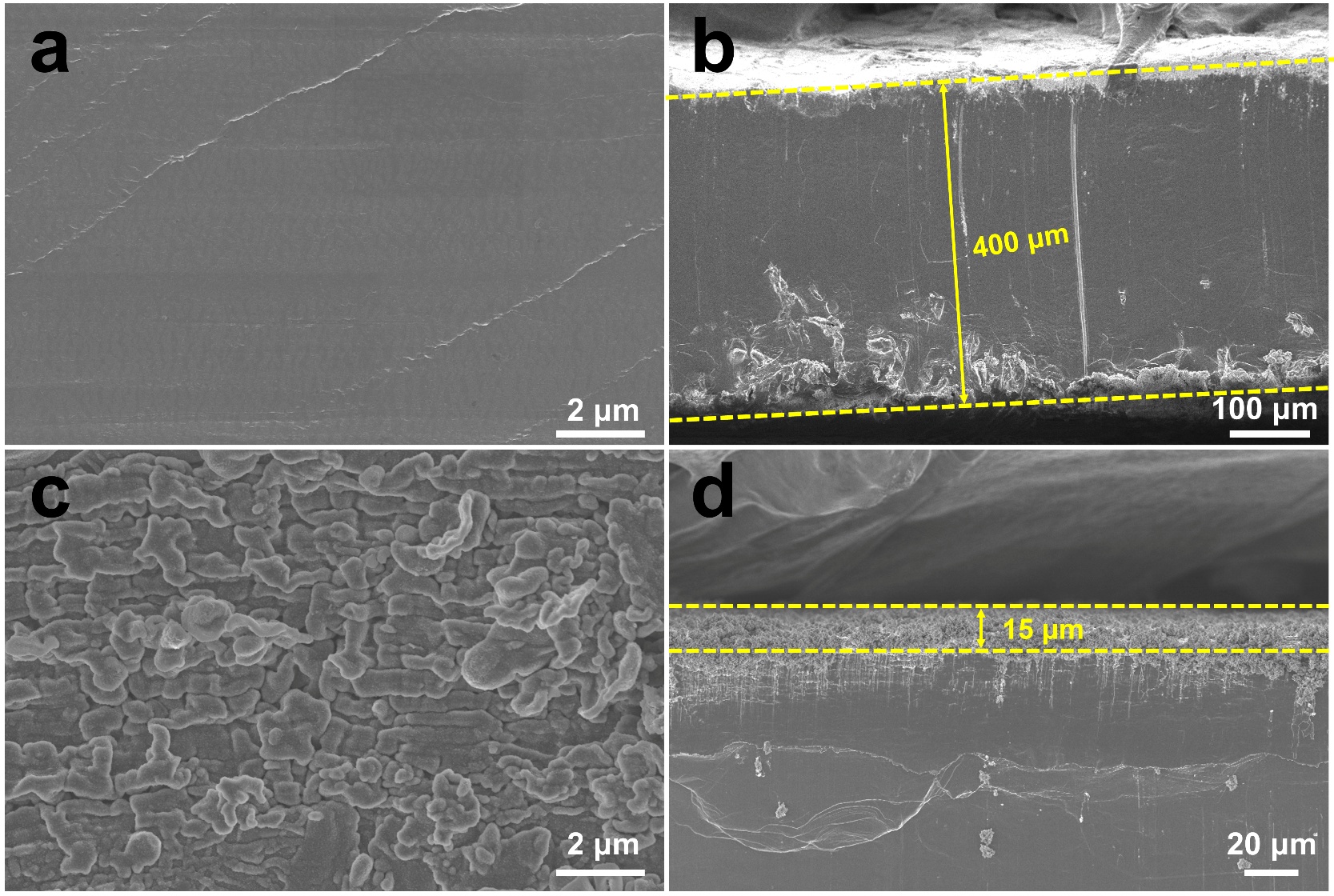


**Fig. S13** The top-view and cross-section SEM images of bare Li (**a, b**) and Z-COF@Li (**c, d**) electrodes before cycling


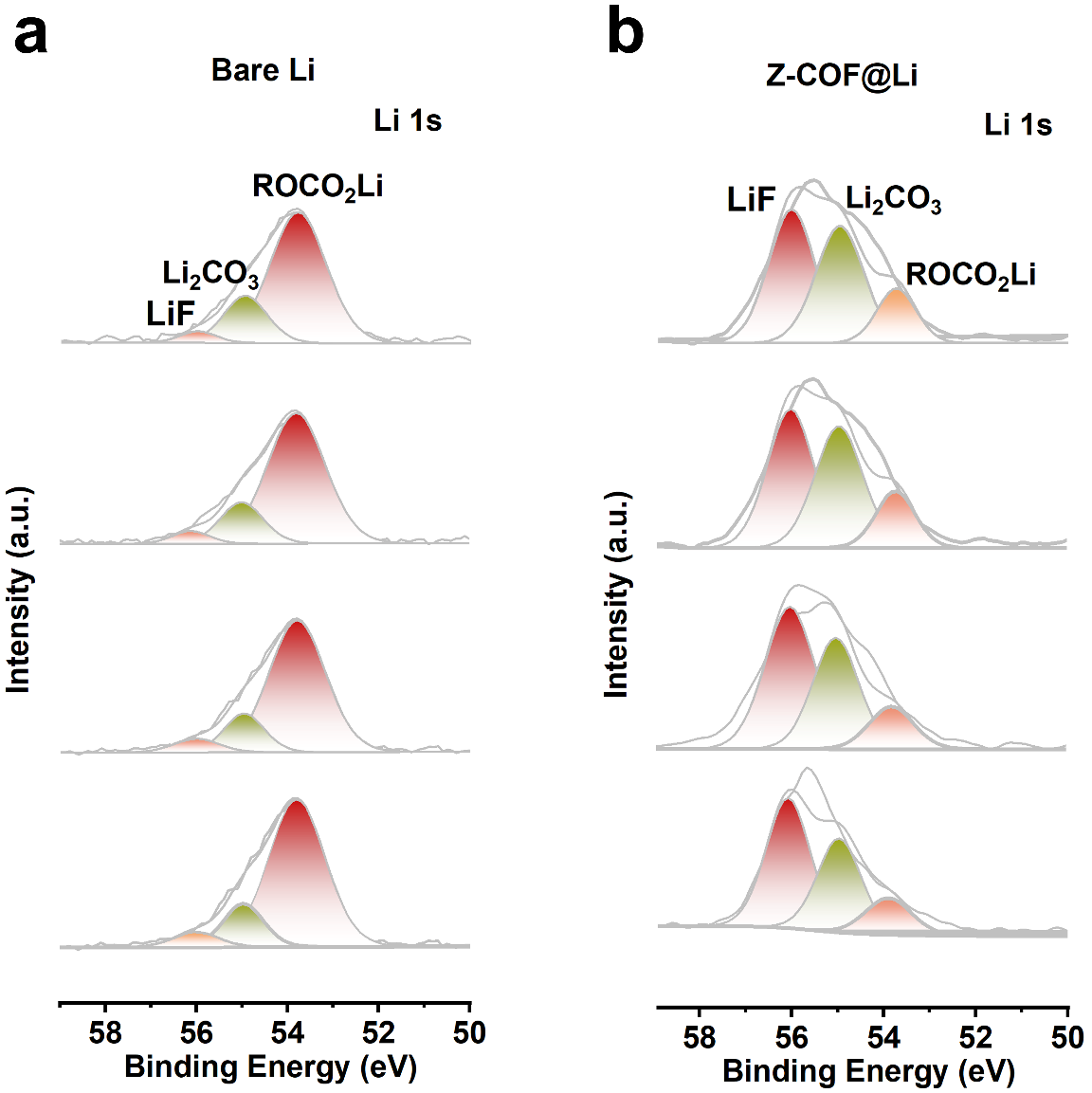


**Fig. S14** The XPS depth profiles of Li 1s spectra in Li metal anodes without (a) and with (b) Z-COF protective layer after 100 cycles


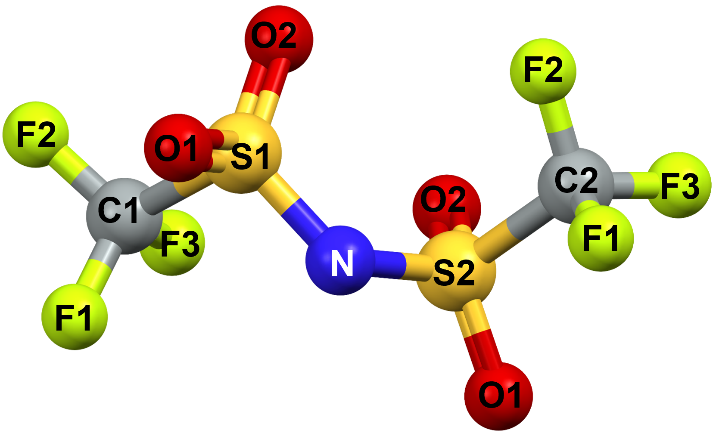


**Fig. S15** DFT optimized geometric model of TFSI^-^


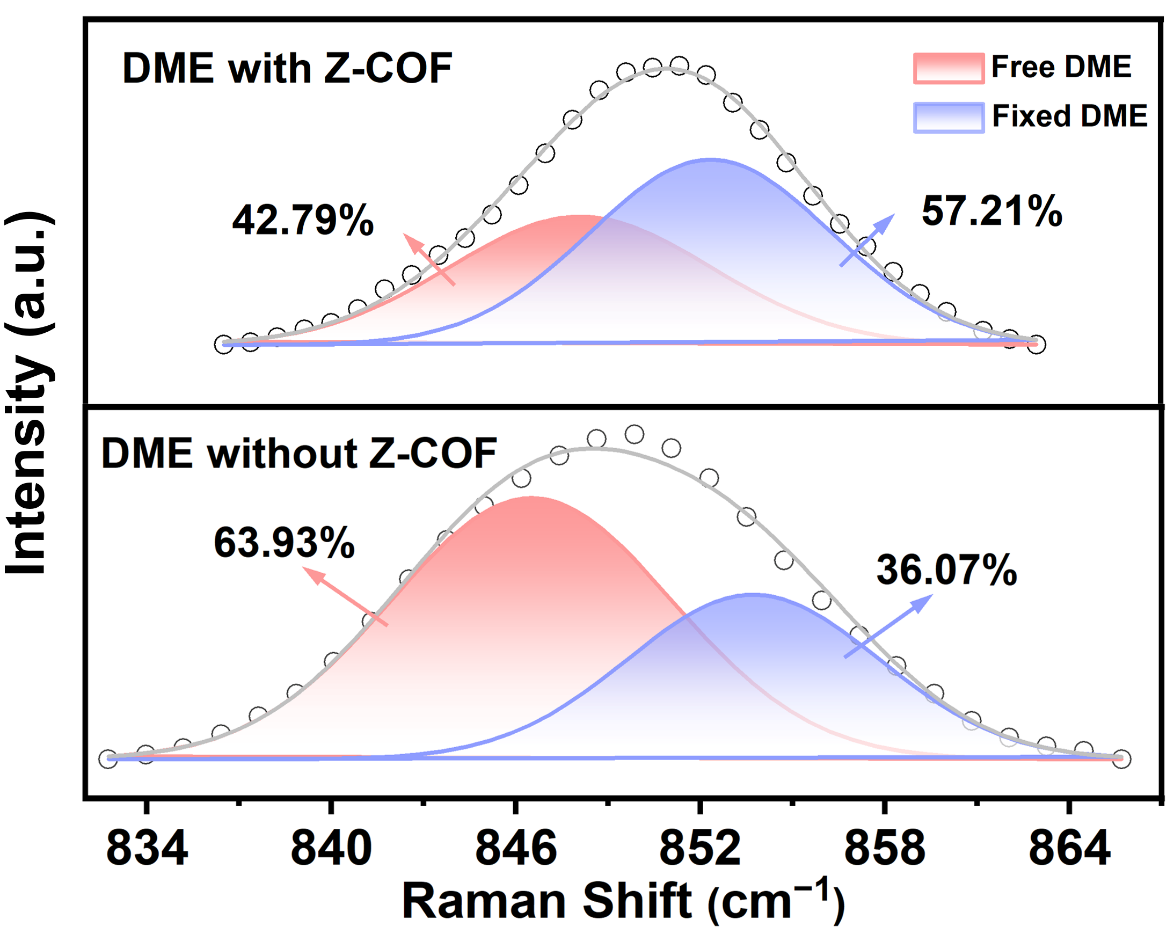


**Fig. S16** Raman spectra of DME with and without Z-COF


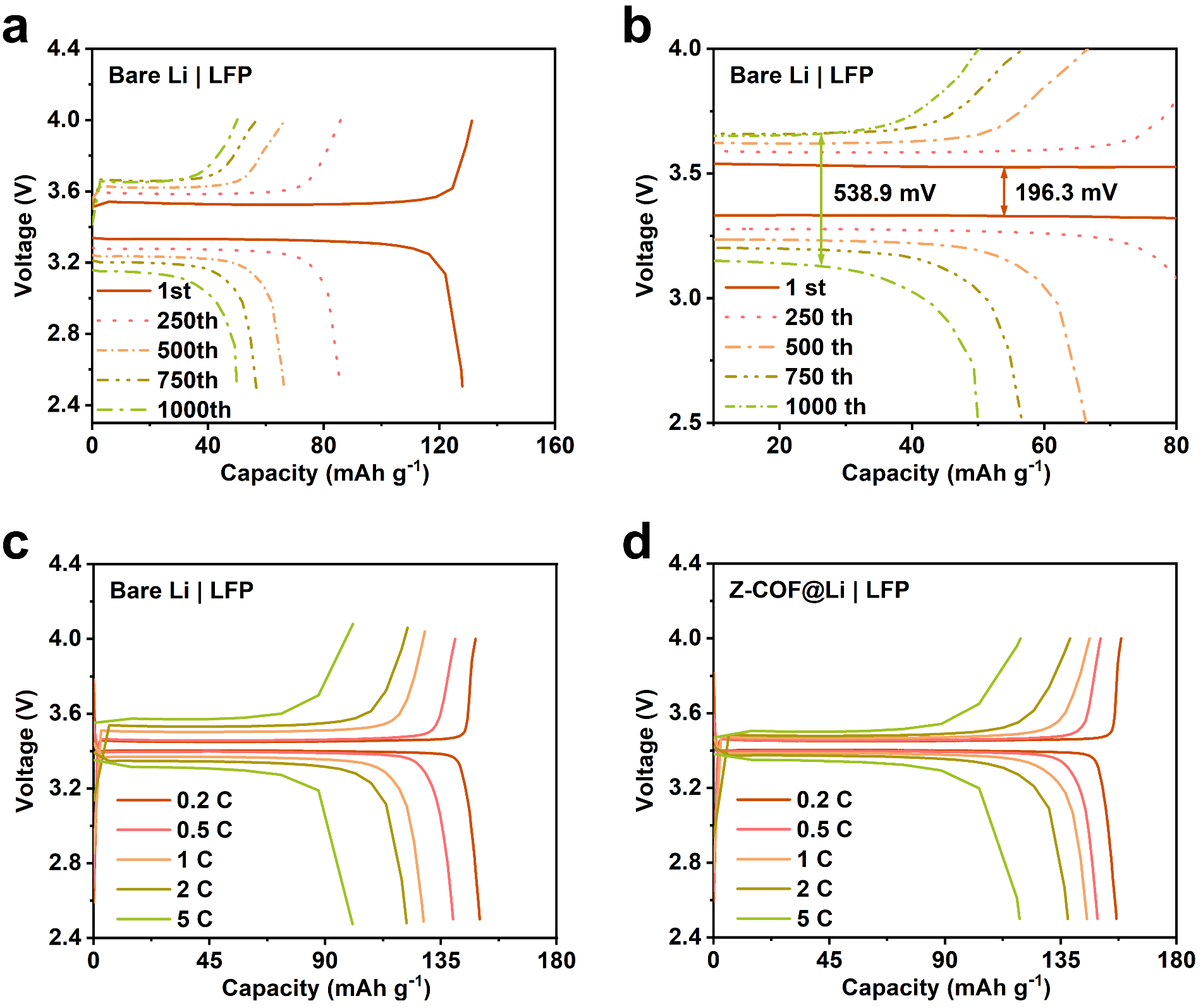


**Fig. S17** (**a**) Galvanostatic charge-discharge curves of bare Li|LFP full cells at 2 C and (**b**) corresponding partial enlargement. Galvanostatic charge-discharge curves of (**c**) Z-COF@Li|LFP and (**d**) bare Li|LFP full cell at various current densities from 0.2 to 5 C, respectively


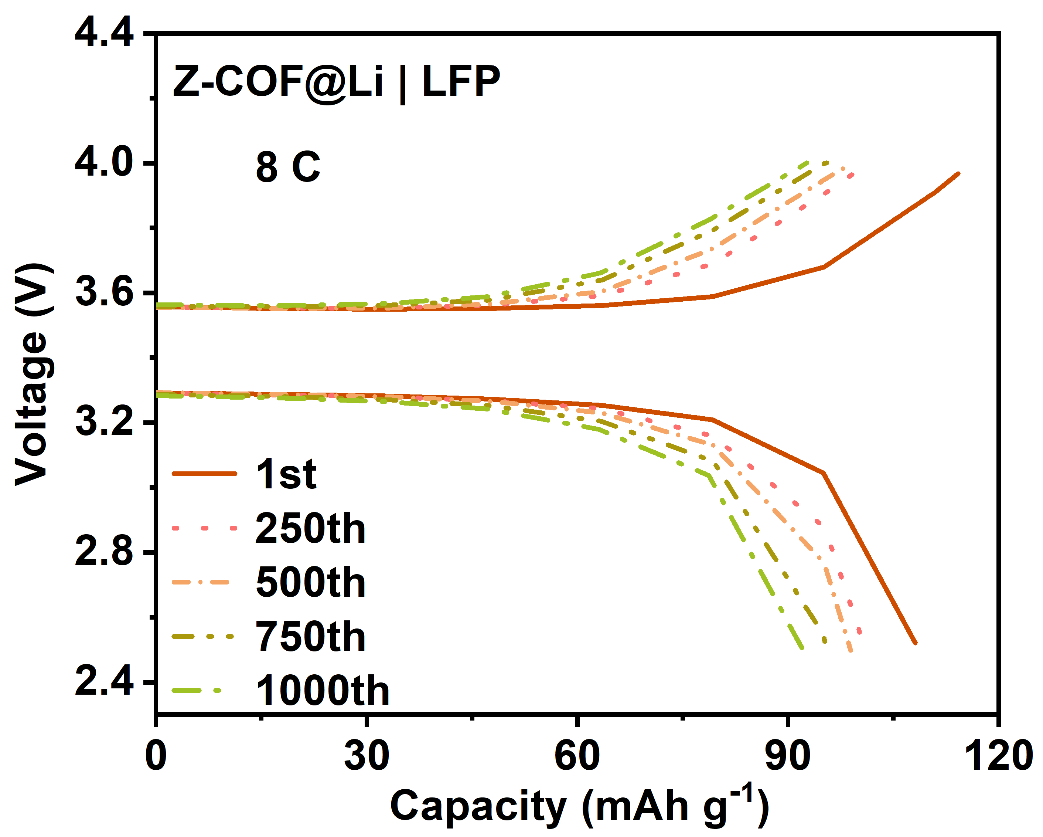


**Fig. S18** Galvanostatic charge-discharge curves of Z-COF@Li|LFP full cells at 8 C


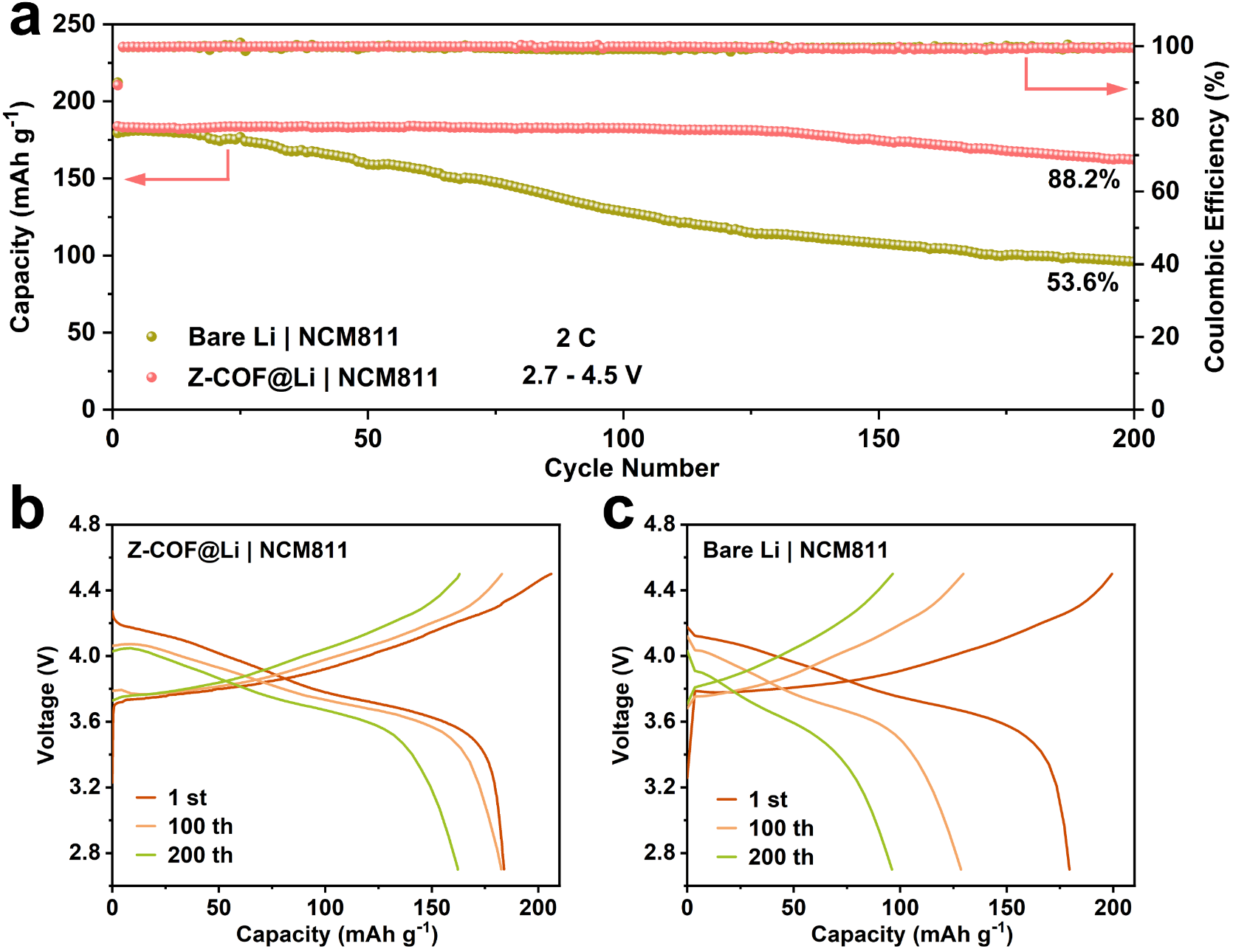


**Fig. S19** (**a**) Long-cycle performance of NCM811 full cells with and without Z-COF protective layer at 2 C. Charge-discharge voltage profiles of (**b**) Z-COF@ Li|NCM811 and (**c**) bare Li|NCM811 cells at 2 C

**Table S1** The N/S ratio (atomic ratio) of Z-COF calculated from XPS

|  | **Z-COF** |
| --- | --- |
| N/S ratio | 3.6 |
| Theoretical value | 4 |

**Table S2** The C, H, N and S elemental analysis of Z-COF (Mass ratio)

|  | N (%) | C (%) | H (%) | S (%) | O (%) |
| --- | --- | --- | --- | --- | --- |
| **Z-COF** | 8.02 | 63.14 | 5.25 | 4.57 | 19.02 |
| Theoretical value | 8.38 | 64.67 | 4.19 | 4.79 | 17.97 |

**Table S3** The Li^+^ transfer number and electrochemical parameters were derived from potentiostatic DC polarization and AC impedance spectra of Li|Li symmetric cells

| **Sample** | **I_0_ (μA)** | **I_s_ (μA)** | **R_0_ (Ω)** | **R_s_ (Ω)** | **ΔV (mV)** | **t_Li_^+^** |
| --- | --- | --- | --- | --- | --- | --- |
| **Bare Li** | 103.8 | 91.2 | 174.9 | 294.8 | 10 | **0.42** |
| **Z-COF@Li** | 125.4 | 117.2 | 143.4 | 158.1 | 10 | **0.87** |

**Table S4** The fitting results of *R*s and *R*_SEI_ at the different temperatures from 303 to 343K according to the equivalent circuit

|  | **Bare Li** | | **Z-COF@Li** | |
| --- | --- | --- | --- | --- |
|  | *R*s | *R*_SEI_ | *R*s | *R*_SEI_ |
| **303K** | 3.9 Ω | 152.3 Ω | 3.6 Ω | 94.4 Ω |
| **313K** | 3.4 Ω | 80.1 Ω | 2.9 Ω | 58.6 Ω |
| **323K** | 2.9 Ω | 46.5 Ω | 2.7 Ω | 34.7 Ω |
| **333K** | 2.8 Ω | 25.5 Ω | 2.6 Ω | 21.8 Ω |
| **343K** | 2.6 Ω | 16.2 Ω | 2.6 Ω | 14.5 Ω |

**Table S5** The C-F and N-S bond length changes in chemical bonds of TFSI^-^ with and without Z-COF

| **Chemical bond** | **TFSI^-^** | | **TFSI^-^@Z-COF** |
| --- | --- | --- | --- |
| C1-F1 | 1.336 | 1.347 | |
| C1-F2 | 1.338 | 1.339 | |
| C1-F3 | 1.338 | 1.342 | |
| C2-F1 | 1.338 | 1.339 | |
| C2-F2 | 1.336 | 1.342 | |
| C2-F3 | 1.346 | 1.346 | |
| Average C-F bond length | 1.339 | 1.343 | |
| N-S1 | 1.621 | 1.625 | |
| N-S2 | 1.619 | 1.621 | |
| Average C-F bond length | 1.620 Å | 1.623Å | |

**Table S6** Cell performance comparison of Z-COF with previous reported Zwitterionic COF materials

| **Materials** | **Cycling Stability (full cell)** | **Capacity retention** | Ionic conductivity (mS cm^−1^) | **Cycling Stability (symmetrical cell)** | t_Li+_ | **References** |
| --- | --- | --- | --- | --- | --- | --- |
| ziCOFNs | Li\|LFP (2 C,700 cycles) | 84.6% | - | 1000 h (1 mA cm^−2^/  1 mA h cm^−2^) | 0.84 | [42] |
| zwitterionic COF | Li\|S (2 C,500 cycles) | 59.4% | 1.0 mS cm^−1^ | 1000 h (1 mA cm^−2^/  1 mA h cm^−2^) | - | [44] |
| Zwitt-COF | Li\|LFP (0.2 C,100 cycles) | - | 0.17 mS cm^−1^ | 100 h (0.3 mA cm^−2^/  1 mA h cm^−2^) | 0.31 | [45] |
| PSZ-COF | Li\|LFP (0.2 C,150 cycles) | - | 0.13 mS cm^−1^ | 300 h (0.1 mA cm^−2^/  1 mA h cm^−2^) | 0.62 | [62] |
| ZW-COF@CNT | Li\|S (1 C,400 cycles) | 81.2% | - | - | - | [63] |
| Z-COF | Li\|LFP (8 C,1000 cycles) | 85.2% | 3.75 mS cm^−1^ | 6300 h (2 mA cm^−2^/  2 mA h cm^−2^) | 0.87 | This work |

**Supplementary References**

1. M. Cui, N. Gao, W. Zhao, H. Zhao, Z. Cao et al., Self-regulating interfacial space charge through polyanion repulsion effect towards dendrite-Free polymer lithium-metal batteries. Adv. Energy Mater. **14,** 2303834 (2024). <https://doi.org/10.1002/aenm.202303834>
2. Z. Ye, S. Zhai, R. Liu, M. Liu, Y. Xu et al., Sulfonate-rich polymer intercalated LDH artificial SEI film to enable high-stability Li-S batteries. Chem. Eng. J. **479**,147847 (2024). <https://doi.org/10.1016/j.cej.2023.147847>
3. P. Zhao, Y. Zhang, B. Sun, R. Qiao, C. Li et al., Song, Enhancing anion-selective catalysis for stable lithium metal pouch cells through charge separated COF Interlayer. Angew. Chem. Int. Ed. **63**, e202317016 (2024) <https://doi.org/10.1002/anie.202317016>
4. P. Hohenberg, W. Kohn, Inhomogeneous Electron Gas. Phys Rev. **136**, B864-B871 (1964). <https://doi.org/10.1103/PhysRev.136.B864>
5. W. Kohn, L.J. Sham, Self-consistent equations including exchange and correlation effects. Phys Rev A. **140**, 1133-1138 (1965). <https://doi.org/10.1103/PhysRev.140.A1133>
6. A.D. Becke, Density-functional exchange-energy approximation with correct asymptotic behavior. Phys Rev A. **38**, 3098-3100 (1988). <https://doi.org/10.1103/PhysRevA.38.3098>
7. T. Lu, F. Chen, Multiwfn: A multifunctional wavefunction analyzer, J. Comput. Chem. **33**, 580-592 (2011). <https://doi.org/10.1002/jcc.22885>
8. J.S. Murray, P. Politzer, The electrostatic potential: an overview. WIREs Comput Mol Sci. **1**, 153-163 (2011). <https://doi.org/10.1002/wcms.19>
9. L. Sheng, L. Wang, J. Wang, H. Xu, X. He, Accelerated lithium-ion conduction in covalent organic frameworks. Chem. Commun. **56**, 10465-10468 (2020). <https://doi.org/10.1039/d0cc04324a>
